# Unraveling Salt Tolerance Mechanisms in Halophytes: A Comparative Study on Four Mediterranean *Limonium* Species with Different Geographic Distribution Patterns

**DOI:** 10.3389/fpls.2017.01438

**Published:** 2017-08-17

**Authors:** Mohamad Al Hassan, Elena Estrelles, Pilar Soriano, María P. López-Gresa, José M. Bellés, Monica Boscaiu, Oscar Vicente

**Affiliations:** ^1^Instituto de Biología Molecular y Celular de Plantas, (UPV-CSIC), Universitat Politècnica de València Valencia, Spain; ^2^Jardín Botánico—ICBiBE, Universitat de València Valencia, Spain; ^3^Instituto Agroforestal Mediterráneo, Universitat Politècnica de València Valencia, Spain

**Keywords:** climate change, ion transport, osmolytes, salinity tolerance, salt glands, salt marsh, salt stress, seed germination

## Abstract

We have performed an extensive study on the responses to salt stress in four related *Limonium* halophytes with different geographic distribution patterns, during seed germination and early vegetative growth. The aims of the work were twofold: to establish the basis for the different chorology of these species, and to identify relevant mechanisms of salt tolerance dependent on the control of ion transport and osmolyte accumulation. Seeds were germinated *in vitro*, in the presence of increasing NaCl concentrations, and subjected to “recovery of germination” tests; germination percentages and velocity were determined to establish the relative tolerance and competitiveness of the four *Limonium* taxa. Salt treatments were also applied to young plants, by 1-month irrigation with NaCl up to 800 mM; then, growth parameters, levels of monovalent and divalent ions (in roots and leaves), and leaf contents of photosynthetic pigments and common osmolytes were determined in control and stressed plants of the four species. Seed germination is the most salt-sensitive developmental phase in *Limonium*. The different germination behavior of the investigated species appears to be responsible for their geographical range size: *L. narbonense* and *L. virgatum*, widespread throughout the Mediterranean, are the most tolerant and the most competitive at higher soil salinities; the endemic *L. santapolense* and *L. girardianum* are the most sensitive and more competitive only at lower salinities. During early vegetative growth, all taxa showed a strong tolerance to salt stress, although slightly higher in *L. virgatum* and *L. santapolense*. Salt tolerance is based on the efficient transport of Na^+^ and Cl^−^ to the leaves and on the accumulation of fructose and proline for osmotic adjustment. Despite some species-specific quantitative differences, the accumulation patterns of the different ions were similar in all species, not explaining differences in tolerance, except for the apparent activation of K^+^ transport to the leaves at high external salinity, observed only in the most tolerant *L. narbonense* and *L. virgatum*. This specific response may be therefore relevant for salt tolerance in *Limonium*. The ecological implications of these results, which can contribute to a more efficient management of salt marshes conservation/regeneration programs, are also discussed.

## Introduction

Halophytes are broadly defined as plants adapted to saline environments that are able to complete their life cycles in the presence of salt concentrations in the soil equivalent to at least 200 mM NaCl (Flowers et al., 1986; Flowers and Colmer, 2008; Santos et al., 2015). Halophytes inhabit different types of saline habitats, both humid and arid, such as littoral and inland salt marshes, dunes, cliffs by the sea, salt lakes, or salt deserts. Salt marshes, in particular, are extremely interesting ecosystems, with great floristic diversity; they have been deteriorated worldwide, due to change of land use for agriculture, urban development, or industrialization. Mediterranean coastal salt marshes suffer, additionally, an intense touristic pressure, which generates profound alterations in the ecosystem, threatening the survival of rare and endemic taxa; they are also very sensitive to the effects of climate change. The conservation value of these areas has been recently highlighted by the EU project “Red List of Habitats” (Janssen et al., 2016). Conservation efforts are mostly focused on recovering the original coverage of structural species, common halophytes that are frequent in salt marshes and other saline habitats and have been studied by many authors (e.g., Koyro, 2006; Redondo-Gómez et al., 2006; Katschnig et al., 2013). Yet the uniqueness of particular salt marshes is largely depending on not-so-common, “differential” taxa including rare, endemic and/or endangered halophytes, which are especially relevant for the biodiversity of these specialized ecosystems but have been much less investigated (Houle et al., 2001; Yıldıztugay et al., 2011; Sekmen et al., 2012).

*Limonium* (Plumbaginaceae) is one of the most biodiverse genera of the Mediterranean halophytic flora, represented in this region by more than 300 species of herbs, shrubs and lianas adapted to saline soils and including many narrow local endemics and threatened taxa (Greuter et al., 1989; Moreno, 2008; Brullo and Erben, 2016). Besides its interest from taxonomic and evolutionary perspectives (Palacios et al., 2000; Lledó et al., 2005), *Limonium* has been the object of physiological and biochemical studies as many species in this genus are highly tolerant to salt stress. *Limonium* belongs to the category of recretohalophytes, plants that secrete salt from their leaves. Salt-excreting structures, which are completely absent in glycophytes, include salt bladders and salt glands. Salt bladders are balloon-like epidermal cells, often modified trichomes, that accumulate large amounts of salt on the leaf surface; salt glands, on the other hand, are stable structures formed by two or more cells that continuously secrete toxic ions to the outside of the plant (Shabala et al., 2014; Yuan et al., 2015, 2016). Epidermal bladder cells are present in about half of all halophytic taxa (Shabala et al., 2014). Salt glands are not so common, appearing on the leaf surfaces of plants belonging to a limited number of plant families, including Plumbaginaceae (Lipschitz and Waisel, 1974).

Secretion of mineral ions by salt glands must be accompanied by efficient osmotic adjustments to avoid dehydration of leaf cells (Shabala et al., 2014). Osmotic balance is maintained predominantly by the accumulation in the cytoplasm of organic compounds acting as compatible solutes or osmolytes. Apart from contributing to osmotic adjustment, osmolytes have additional functions in stress tolerance mechanisms, directly protecting macromolecular structures under stress conditions—in their role as low-molecular-weight chaperons—and also as scavengers of “reactive oxygen species” (ROS) or as signaling molecules (Flowers and Colmer, 2008; Munns and Tester, 2008; Türkan and Demiral, 2009; Szabados and Savouré, 2010; Slama et al., 2015). However, osmolyte biosynthesis represents a high cost for the plants, since the same cellular osmolarity can be reached by ion uptake and transport with much lower energy consumption (Raven, 1985; Shabala and Shabala, 2011). Therefore, osmotic adjustment is also achieved through the accumulation of inorganic ions, such as Na^+^ and Cl^−^, which are primarily stored in the vacuoles to avoid their toxic effects in the cytosol, according to the “ion compartmentalization hypothesis” (Wyn Jones et al., 1977; Yeo, 1983; Glenn et al., 1999). This response to salt stress is not specific for recretohalophytes but common to all salt-tolerant plants and is especially efficient in dicotyledonous succulent halophytes, which possess swollen vacuoles capable of storing large amounts of salt. In monocot halophytes, on the other hand, salt tolerance is mostly based on mechanisms blocking transport of toxic ions from the roots to the aerial part of the plants (Briens and Larher, 1982; Tipirdamaz et al., 2006; Gil et al., 2014).

Salt tolerance of a given species is largely dependent on the plant developmental stage, older plants being usually more resistant than younger ones (Johnson et al., 1992; Houle et al., 2001; Vicente et al., 2004). Seed germination and seedling establishment are generally the most sensitive phases of the life cycle for adaptation to saline soils; only those plants that overcome this bottleneck will shape natural plant communities in a territory (Rubio-Casal et al., 2001; Donohue et al., 2010).

In this paper, we present an extensive study on the responses to controlled salt treatments of four Mediterranean *Limonium* species that, although closely related taxonomically, have different distribution patterns in nature: a narrow local endemism, a second endemic species with a wider distribution, and two taxa present throughout the Mediterranean basin. Our working hypothesis, based on the aforementioned data, was that the geographic range size and distribution of the selected *Limonium* species are dependent on their relative degree of salt tolerance at the seed germination and/or early vegetative growth stage(s). Therefore, one of the specific aims of the work was to check this by correlating the relative salinity tolerance of the four taxa—estimated from the inhibition of seed germination and seedling growth in the presence of increasing NaCl concentrations—with the extension and characteristics of their natural habitats.

On the other hand, performing comparative analyses of the responses to salt stress of genetically related taxa differing in their stress resistance is a useful strategy to decipher salt tolerance mechanisms in those species. In this case, the basic assumption is that those responses that are important for tolerance should be more efficiently activated in the more tolerant species. Accordingly, an additional aim of our study was to identify the most relevant mechanisms of salt tolerance in *Limonium*, by correlating the activation of specific responses to salt stress—focusing on the control of ion transport and osmolyte accumulation—with the relative tolerance of the investigated species during early vegetative growth.

## Materials and methods

### Plant material

Four perennial hemicryptophyte species of the genus *Limonium*, section *Limonium*, were selected for this study. They grow in salt marshes of the east coast of the Iberian Peninsula, but with different geographical distribution and range sizes. *L. santapolense* Erben is a local endemic of the Alicante province, included in the Spanish red list of vascular flora under the UICN category of “vulnerable,” due to its risk of extinction (Moreno, 2008). *L. girardianum* (Guss.) Fourr. is endemic to the Mediterranean coastal salt marshes of southern France and eastern Spain, with highly fragmented distribution areas. This species is also threatened by habitat transformation and it is protected in France (Baumberger et al., 2012). *L. virgatum* (Willd.) Fourr. and *L. narbonense* Mill. have a wider distribution area, mainly throughout the northern coasts of the Mediterranean. Data on species distribution areas were obtained from the Global Biodiversity Information Facility (GBIF Secretariat, 2016).

### Field sites description

Seeds of the four selected species were collected at the time of natural dispersal, from natural habitats in two protected areas located on the eastern coast of Spain: “Clot de Galvany” in the Alicante province (*L. santapolense*) and “Devesa de El Saler” in the Valencia province (*L. girardianum, L. narbonense* and *L. virgatum*). “Clot de Galvany” covers 366 hectares of a coastal marsh area linked to a set of flooded lands and sandy dunes. It was designated as Municipal Natural Park (MNP) by the local government and also as Special Protection Area (SPA) and Site of Community Importance (SCI) by the Nature 2000 network. The “Devesa de El Saler” is included in “La Albufera” Natural Park, one of the most important protected areas in Spain, also considered as Nature 2000 SPA and SCI. It covers 21,120 hectares, which are distributed in a shallow saltwater body, a sand bar and a Mediterranean pine forest with rich shrubbery growing in sandy soil. Both of these areas are characterized by a Mediterranean climate with dry summer periods alternating with torrential raining episodes, mainly in autumn and some years also in spring. Mean temperature for “Clot de Galvany” is 18.2°C and yearly rainfall 317 mm, and for “Devesa de El Saler” 17.6°C and 443 mm; these data were obtained from the closest meteorological stations, in Santa Pola (Alicante) and Silla (Valencia), respectively (https://es.climate-data.org/).

### Germination assays

Collected seeds were cleaned and stored in paper bags in a controlled environment, at 20°C and 40–50% RH, until used for the experiments. *In vitro* germination assays were performed using four replications per species and per treatment, each with 25 seeds placed on 0.6% agar in 55 mm-diameter Petri dishes, which were kept in climate-controlled cabinets. Tests were performed at 30°C—the temperature established from previous *in vitro* germination assays—which is in accordance with values recorded in the field. Illumination was provided with daylight fluorescent tubes, under 12 h photoperiod and a mean irradiance of 100 μmol·m^−2^·s^−1^.

Salt stress effects on germination were tested at increasing NaCl concentrations: 0, 50, 100, 150, 200, 300, 400, and 500 mM. No seeds of any of the studied species germinated at higher salt concentrations in preliminary tests. The final germination percentage (GP) was determined after 30 days, and mean germination time (MGT) was calculated according to Ellis and Roberts (1981), to assess the rate of germination. MGT was not calculated if germination percentages were ≤ 5%. To better compare the behavior of the different species regarding salt-induced inhibition of germination, “reduction of germination percentage” was calculated as: RGP = [1−(N° of germinated seeds in the salt treatment/N° of germinated seeds in the control)] × 100 (Madidi et al., 2004). All data in tables and graphs are expressed as the means of the four replications, with standard errors (SE).

Seeds that did not germinate in the presence of NaCl in the previous assays were included in recovery tests. Seeds were briefly rinsed and transferred to distilled water and then maintained at the same temperature and light regimes mentioned above, to check their recovery capacity. No germination was observed after 10 days of incubation and the recovery tests were stopped at day 15. The “recovery of germination” percentages were calculated following Zaman et al. (2010).

Seed germination responses to water potential were described by hydrotime analysis, as:

$$\begin{array}{l} \theta \text{H}=\left[\text{Ψ}-\Psi \text{b}(\text{g})\right]\text{tg} \end{array}$$

where Ψ is the water potential of the external medium (MPa), Ψb(g) is the base water potential (the minimum water potential permitting germination) defined for a specific germination fraction g, and tg is the time required for germination of percentage g (Gummerson, 1986; Bradford, 1990). The base water potential (Ψb) for the four species was calculated by extrapolating the least-squares regression line on the plot of 1/MGT against the osmotic potential to the x-axis intercept. Hydrotime (θ) was estimated as the inverse of the slope of these regression lines (Kebreab and Murdoch, 1999). The osmotic potentials (Ψ) of the different salt concentrations were calculated according to the Van't Hoff equation for tests at 30°C (Ben-Gal et al., 2009).

### Plant growth and salt treatments

Seeds were sown without prior sterilization, on a mixture of commercial peat and vermiculite (3:1) and watered twice a week with Hoagland nutritive solution (Hoagland and Arnon, 1950). After 3 weeks, seedlings of the same size (for each species) were selected and placed individually in one-liter pots. After a few days of acclimatization in their new containers, salt treatments were initiated by watering the plants once every 5 days with aqueous NaCl solutions at 200, 400, or 800 mM final concentrations, or with water for the non-stressed controls; these solutions were added to the trays containing the pots (1 L per tray, with 5 pots each). Five individual plants were used as biological replicas per species and per treatment. After 1 month, plant aerial parts (rosette leaves) and roots were harvested for further studies; substrate samples of the pots were collected at the same time. All experiments were conducted in a controlled environment chamber, under the following conditions: long-day photoperiod (16 h of light/8 h of darkness), temperature of 23°C during the day and 17°C at night, and 50–80% relative humidity.

### Soil analysis

The electrical conductivity of the substrate in 1-to-5 suspensions (EC_1:5_) was checked at the end of the treatments. Substrate samples, taken from the five pots used per treatment, were air-dried and then passed through a 2 mm sieve. A soil:water (1:5) suspension was prepared in distilled water and stirred for 1 h at 600 rpm and 21°C. EC was measured with a Crison Conductivity meter 522 (Crison Instruments SA, Barcelona, Spain) and expressed in dS m^−1^.

For comparison with natural conditions, soil samples were collected from the same locations as the seeds during summer, when salinity is higher due to the specific Mediterranean climatic conditions (Gil et al., 2014). Analysis of the EC_1:5_ of the soil was carried out as for the pot substrates from the greenhouse, with five independent samples taken from each collection site.

### Collection of plant material and plant growth parameters

To assess the effects of salt stress on *Limonium* plants at the stage of vegetative growth, the following parameters were determined at the end of the treatments, in the leaves of control and salt-stressed plants: fresh weight (FW), dry weight (DW), water content percentage (WC%), and leaf surface area (LA). After weighing the whole aerial part of the harvested plants, five rosette leaves were selected from each of them and scanned; ImageJ software (Rasband, 1997-2012) was used to measure LA. The remaining leaves were thoroughly rinsed with deionized water (to eliminate salt on the leaf surface), blotted dry on filter paper and stored for further analyses. To better compare the effects of salt treatments on the different *Limonium* species—which differ in plant size—DW and LA were expressed as percentages of the mean values determined for the corresponding controls, taken as 100%. Absolute values of DW and LA were the following: 0.77 g and 17.01 cm^2^ (*L. santapolense*), 0.29 g and 4.45 cm^2^ (*L. virgatum*), 0.32 g and 6.06 cm^2^ (*L. girardianum*), 0.30 g and 8.60 cm^2^ (*L. narbonense*), respectively. To determine water content, a fraction of each leaf sample was weighed (FW), dried until constant weight at 65°C (48–72 h), and then weighed again (DW); WC% was calculated as: [(FW-DW)/FW] × 100 (Gil et al., 2014). Fresh plant material was frozen in liquid N_2_ and stored at −75°C, and dry material was kept in tightly closed tubes at room temperature.

Total root FW could not be measured, as the root system of *Limonium* plants included a mass of very thin roots that broke when the plants were uprooted. The root fraction which was recovered from each plant was thoroughly cleaned with a paintbrush, briefly washed with deionized water and blotted dry on filter paper, and the FW, DW, and WC% were determined as for the aerial part.

### Photosynthetic pigments

Chlorophylls a and b (Chl a, and Chl b) and total carotenoids (Caro), were determined as described by Lichtenthaler and Wellburn (1983). Fresh leaf material (50 mg) was extracted with 10 mL ice cold 80% (v/v) acetone by gently mixing overnight in an orbital shaker. The sample was centrifuged 10 min at 12,000 rpm, and the absorbance of the supernatant was measured at 663, 646, and 470 nm. Chl a, Chl b, and Caro concentrations were calculated using the equations in Lichtenthaler and Wellburn (1983), and pigment contents were expressed in mg g^−1^ DW.

### Ion content measurements

Ion contents in roots and leaves were determined according to Weimberg (1987), in aqueous extracts obtained by heating the samples (50 mg of dried, ground plant material in 15 mL of water) for 1 h in a boiling water bath, followed by filtration through a 0.45 μm nylon filter. The five leaves per harvested plant previously selected to determine LA were used to recover ions on the leaf surface, by immersing each leaf in 15 ml MilliQ water for 10 min; dissolved ions were directly measured in the solution and their contents were expressed as μmol per cm^2^ of LA. This simple method, based on Tabot and Adams (2014), allows quantification of the total amount of salt accumulated on the surface of the leaves, although not the secretion rate of salt glands; yet it is useful for comparative analyses of related species subjected to the same salt treatments, as those described in this paper.

Monovalent cations (Na^+^ and K^+^) were quantified with a PFP7 flame photometer (Jenway Inc., Burlington, VT, USA), chlorides were measured with a chloride analyzer, and divalent cations (Ca^2+^ and Mg^2+^) using an atomic absorption spectrometer SpectrAA 220 (Varian Inc., CA, USA).

### Osmolyte quantification

Free proline (Pro) was extracted from 50 mg of dry leaf material with 2 mL of a 3% (w/v) aqueous sulfosalicylic acid solution and was quantified according to Bates et al. (1973). The extract was mixed with acid ninhydrin, incubated for 1 h at 95°C and, after cooling on ice, extracted with toluene. The absorbance of the organic phase was determined at 520 nm, with toluene as a blank. Pro concentrations were expressed as μmol·g^−1^ DW.

Glycine betaine (GB) was determined in aqueous leaf extracts (50 mg dry leaf material in 1 mL MilliQ water), according to Grieve and Grattan (1983) with the modifications proposed by Nawaz and Ashraf (2010). The sample was mixed with potassium iodide, incubated on ice for 90 min and extracted with 1, 2-dichlorethane (pre-cooled at −20°C); the absorbance of the solution was finally measured at 365 nm. GB concentration was expressed as μmol·g^−1^ DW.

Total soluble sugars (TSS) contents were determined following the method of Dubois et al. (1956). Dry leaf material (50 mg) was ground and mixed with 3 ml of 80% (v/v) methanol on a rocker shaker for 24 h, and the extract was recovered by centrifugation; concentrated sulfuric acid and 5% phenol was added to the supernatant and the absorbance was measured at 490 nm. TSS contents were expressed as “mg equivalent of glucose” per g DW.

### HPLC analysis of soluble carbohydrates

The water-soluble sugar fraction was analyzed using a Waters 1,525 HPLC system coupled to a 2,424 evaporative light scattering detector (ELSD). The source parameters of ELSD were the following: gain 75, data rate 1 point per second, nebulizer heating 60%, drift tube 50°C, and gas pressure 2.8 Kg/cm^2^. Plant dry material (50 mg) was boiled in 10 ml MilliQ water for 10 min and then filtered through 0.22 μm nylon filters. Twenty microliters of aliquots were injected into a Prontosil 120-3-amino column (4.6 × 125 mm; 3 μm particle size) maintained at room temperature, using a Waters 717 autosampler. In each run, an isocratic flux of 85% acetonitrile was applied for 25 min at 1 mL/min. Fructose (Fru) and sucrose (Suc) were identified as the major carbohydrates in the samples and were quantified by peak integration using the Waters Empower software and comparison with the corresponding purified standards.

### Statistical analysis

Germination responses were statistically assessed using the SPSS v. 15.0 software (SPSS Inc, Chicago, IL, USA). The significance (*p* < 0.05) of differences among treatments was tested by applying one-way ANOVA according to Khan and Rayner (2003). Percentage germination values were arcsine transformed prior to the statistical analysis. Analysis of variance was carried out separately on data from each species. For the salinity tests, when the ANOVA null hypothesis was rejected, differences between salt treated groups and the controls were evaluated by Dunnett's *t*-test. To find the variables with the greatest influence on species response, a nonlinear principal component analysis was applied (NLPCA; de Leeuw, 1982) using the program CATPCA (included in the SPSS software package). This kind of analysis incorporates also categorical variables and allows discovering nonlinear relationships between them. Moreover, Alpha of Cronbach was calculated (Cronbach, 1951) for each extracted component. If this value is high for a specific component, it would be interpreted as an indicator of the weight of the component. In addition, it serves to explain the total variance. In general, alpha values equal to or >0.7 are considered reliable (Bland and Altman, 1997).

Growth and biochemical parameters were analyzed using the program Statgraphics Centurion v. XVI (Statpoint Technologies Inc., Warrenton, VA, USA). Before the analysis of variance, the Shapiro–Wilk test was used to check for validity of normality assumption and Levene test for the homogeneity of variance. If ANOVA requirements were accomplished, the significance of the differences between treatments (for each species), and between species (for each treatment) was independently tested by one-way ANOVA at a 95% confidence level; *post-hoc* comparisons were made using the Tukey HSD test. In addition, all variables determined in plants submitted to salt stress treatments were correlated by principal component analysis (PCA), independently for each of the four studied *Limonium* species. Each principal component was a linear combination of the original variables with coefficients equal to the eigenvectors of the correlation matrix. The results of these analyses were represented graphically, for each species, as a biplot of the two main principal components.

A two-way ANOVA, including all measured variables—in the seed germination assays and the salt treatments of the plants—was also carried out to evaluate the significance of differences between species, between treatments and for the interaction species × treatment.

## Results

### *In vitro* germination assays

**Figure 1:**
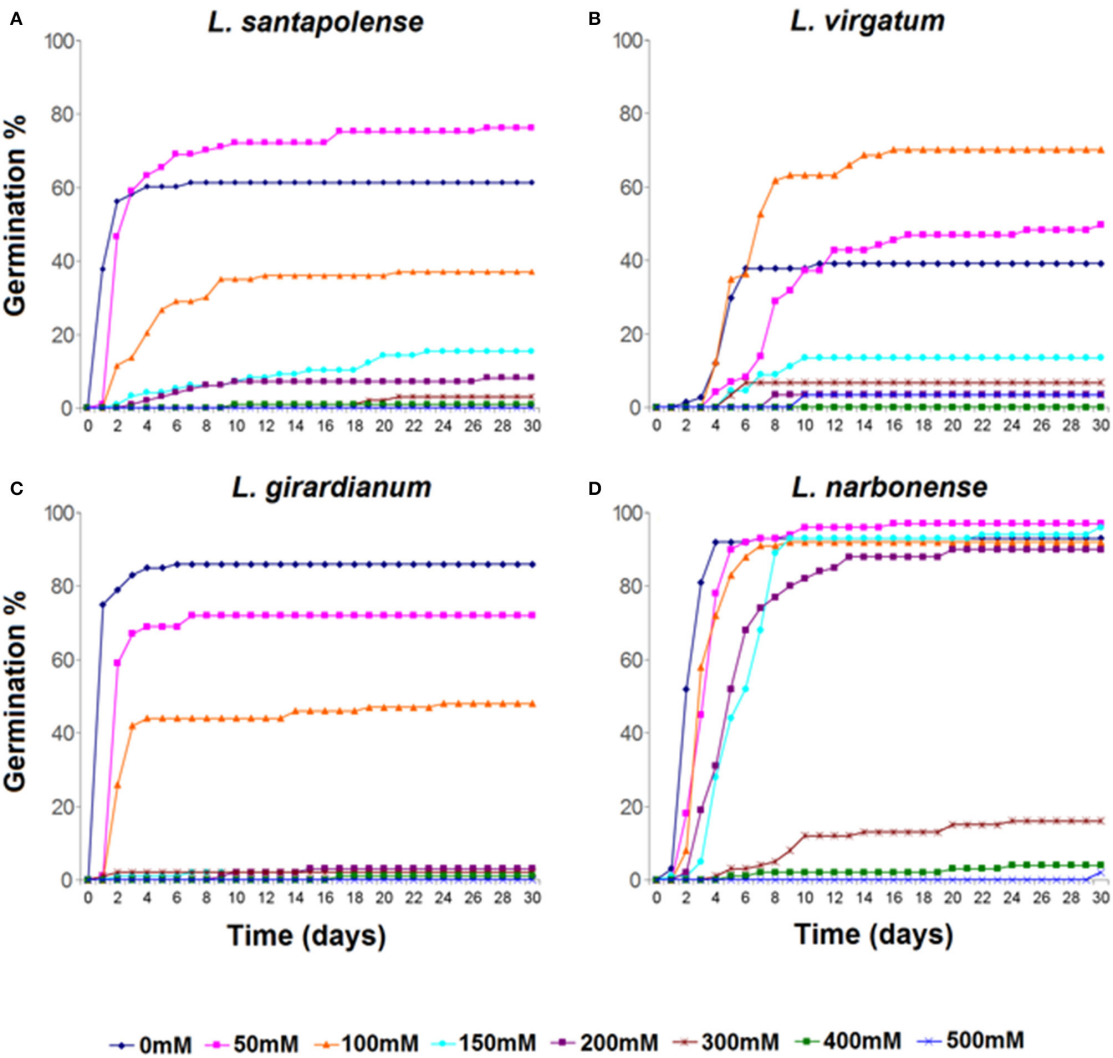
Cumulative seed germination percentages of *Limonium* species during 30 days of incubation in the presence of the indicated NaCl concentrations. **(A)** *L. santapolense*; **(B)** *L. virgatum*; **(C)** *L. girardianum*; **(D)** *L. narbonense*.

**Figure 2:**
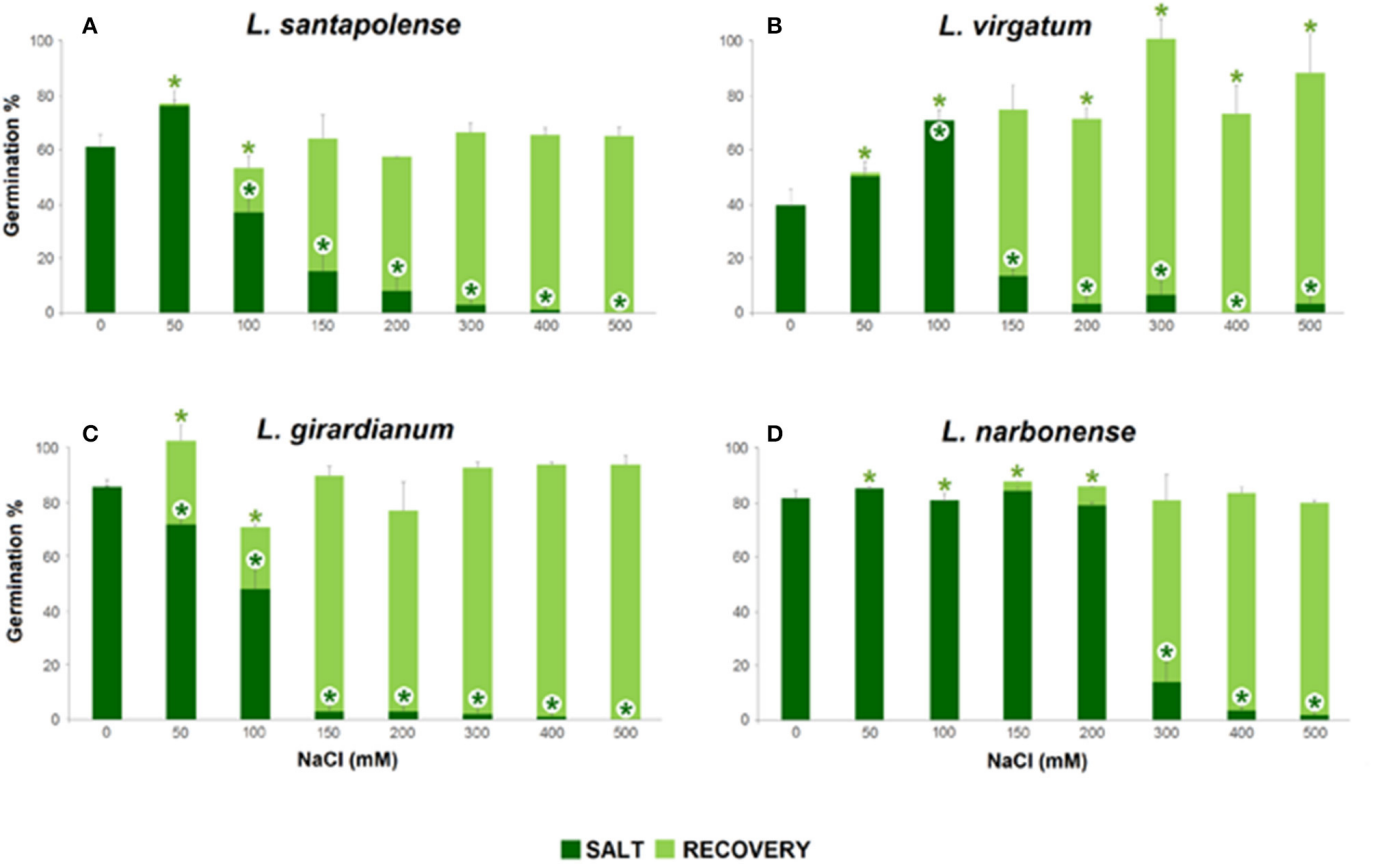
Total germination percentages after 30 days at the indicated salt concentrations, showing the salinity effect (dark green), and total germination percentages after 15 days of “recovery” (light green), in the four studied *Limonium* species. **(A)** *L. santapolense*; **(B)** *L. virgatum*; **(C)** *L. girardianum*; **(D)** *L. narbonense*. For recovery, seeds were transferred from each NaCl solutions to distilled water. Asterisks indicate significant differences in each condition respect to the corresponding control (according to Dunnet test, *p* < 0.05).

High NaCl concentrations inhibited seed germination in all tested *Limonium* species, but with remarkable differences regarding both, progression of germination and final germination percentages (Figures 1, 2). Germination under control conditions (in water) varied from about 40% in *L. virgatum* or 60% in *L. santapolense*, to more than 80% in *L. girardianum* and *L. narbonense*. The salt-dependent reduction of germination percentages also differed between species. According to this criterion, *L. narbonense* appeared to be the most salt tolerant: NaCl up to 200 mM did not inhibit germination, and a significant reduction was only observed at concentrations of 300 mM and above (Figures 1D, 2D). The most salt-sensitive taxon would be *L. girardianum*, which showed a progressive inhibition, with significant reductions even at 50 mM NaCl (Figures 1C, 2C). Intermediate degrees of tolerance were observed in *L. virgatum* (Figures 1B, 2B) and *L. santapolense* (Figures 1A, 2A). A significant *stimulation* of germination was detected at low NaCl concentrations in *L. virgatum* (ca. 22% increase at 50 mM and 80% at 100 mM; Figures 1A, 2A) and *L. santapolense* (25% at 50 mM; Figures 1B, 2B). As mentioned above, these are the two taxa showing lower germination in the controls.

Seeds which did not germinate in the presence of NaCl were transferred to distilled water, and germination percentages were calculated again, after 15 days. Salt pre-treatments—even at the highest concentration tested—did not affect germination capacity of the seeds. Full recovery was observed in all *Limonium* taxa: total germination percentages reached, at least, the same values than for control treatments in the absence of salt (Figure 2). Moreover, previous incubation of *L. virgatum* seeds with ≥150 mM NaCl resulted in a significant stimulation of germination (Figure 2B); the same was observed, to a lesser extent, for *L. girardianum* seeds pre-treated with 50 mM NaCl (Figure 2C).

Germination velocity was also determined, as an additional criterion to evaluate salt tolerance, by calculating the “mean germination time” (MGT) for each treatment (Table 1). A general increase of MGT with increasing NaCl concentrations was observed, indicating a reduction in germination velocity. Variations in MGT between species roughly correlated with their relative salt tolerance, estimated from the reduction of final germination percentages. Thus, MGT increase was more pronounced in *L. girardianum* (the most salt-sensitive taxa), followed by *L. santapolense*, while the least affected was *L. narbonense*; no clear trend was detected in *L. virgatum* (Table 1). Seed germination was generally faster in the “recovery” experiments after exposure to salt than in the initial germination tests. Germination velocity increased with respect to the controls in *L. virgatum* and *L. santapolense* for all tested concentrations, as indicated by lower MGT values (Table 1). Seeds of *L. narbonense* also showed a more rapid germination than in the controls, but only after pre-treatments with ≥300 mM NaCl; this priming effect of salt, however, was not observed in *L. girardianum* (Table 1).

**Table 1:** Mean germination time (MGT) of seeds during the initial germination tests in the presence of the indicated salt concentrations, and during recovery after transferring the salt-treated seeds to distilled water.

**MGT**								
**NaCl (mM)**	**Seed germination**				**Recovery**			
	***Ls***	***Lv***	***Lg***	***Ln***	***Ls***	***Lv***	***Lg***	***Ln***
0	0.9 ± 0.2	4.8 ± 0.4	0.4 ± 0.2	2.6 ± 0.1				
50	3.9 ± 0.7	10.2 ± 1.5^*^	2.3 ± 0.1^*^	3.8 ± 0.3	−	−	3.0 ± 0.2^*^	−
100	5.3 ± 0.7^*^	6.6 ± 0.2	3.9 ± 0.7^*^	3.7 ± 0.1	0.0 ± 0.0^*^	−	2.8 ± 0.2^*^	−
150	11.3 ± 1.0^*^	6.7 ± 0.9	−	6.6 ± 0.3^*^	0.0 ± 0.0^*^	0.4 ± 0.3^*^	0.7 ± 0.4	−
200	7.9 ± 1.9^*^	−	−	5.9 ± 0.6^*^	0.1 ± 0.1^*^	0.0 ± 0.0^*^	1.9 ± 0.1^*^	3.0 ± 0.0
300	−	−	−	10.5 ± 2.4^*^	0.0 ± 0.0^*^	0.5 ± 0.4^*^	0.6 ± 0.1	0.6 ± 0.1^*^
400	−	−	−	−	0.3 ± 0.1^*^	0.0 ± 0.0^*^	0.1 ± 0.1	0.3 ± 0.2^*^
500	−	−	−	−	0.1 ± 0.0^*^	0.2 ± 0.2^*^	0.0 ± 0.0	0.3 ± 0.0^*^

Ls, Limonium santapolense; Lv, L. virgatum; Lg, L. girardianum; Ln, L. narbonense. Asterisks (^*^) indicate significant differences in each condition respect to the control (Dunnet test, p < 0.05).

Two-way ANOVA showed significant differences for the variables “species,” “treatment” and their interaction, concerning germination percentages and MGT, both in the initial germination tests and the “recovery” experiments (Table 2).

**Table 2:** Two-way ANOVA.

**Parameter**	**A—Species**	**B—Treatment**	**A × B—Interaction**
GP	0.0000^***^	0.0000^***^	0.0000^***^
MGT	0.0240^*^	0.0000^***^	0.0000^***^
GPrec	0.0000^***^	0.0000^***^	0.0000^***^
MGTrec	0.0000^***^	0.0000^***^	0.0000^***^
EC_1:5_	0.0000^***^	0.0367^*^	0.001^**^
Leaf DW	0.3153^ns^	0.0516^ns^	0.4606^ns^
Leaf Area	0.0000^***^	0.0005^***^	0.2953^ns^
WC% (leaves)	0.0238^*^	0.0000^***^	0.6598^ns^
WC% (roots)	0.0000^***^	0.002^**^	0.0008^***^
Chl a	0.0006^***^	0.0000^***^	0.2631^ns^
Chl b	0.2106^ns^	0.0000^***^	0.934^ns^
Caro	0.0039^**^	0.0001^***^	0.2192^ns^
Na^+^ roots	0.0000^***^	0.0000^***^	0.0033^**^
Cl^−^ roots	0.0000^***^	0.0000^***^	0.0278^*^
K^+^ roots	0.0000^***^	0.0000^***^	0.0056^**^
Ca^2+^ roots	0.0000^***^	0.0037^**^	0.0000^***^
Mg^2+^ roots	0.0000^***^	0.0859^ns^	0.0262^*^
Na^+^ leaves	0.6838^ns^	0.0000^***^	0.7859^ns^
Cl^−^ leaves	0.0000^***^	0.0000^***^	0.1542^ns^
K^+^ leaves	0.0013^**^	0.0000^***^	0.0018^**^
Ca^2+^ leaves	0.0000^***^	0.4435^ns^	0.227^ns^
Mg^2+^ leaves	0.0000^***^	0.2719^ns^	0.2495^ns^
Pro	0.0000^***^	0.0000^***^	0.0000^***^
GB	0.0000^***^	0.0000^***^	0.0000^***^
TSS	0.0000^***^	0.0000^***^	0.0000^***^
Fru	0.0000^***^	0.0000^***^	0.0000^***^
Suc	0.0000^***^	0.0000^***^	0.0000^***^

*Effect of the variables “species” and “treatments,” as well as the interaction “species × treatment” on all measured parameters, as established by two-way ANOVA. Values represent P and its statistical significance. GP, germination percentage; MGT, mean germination time; GPrec, germination percentage in “recovery” tests; MGTrec, mean germination time in “recovery” tests; EC_1:5_, substrate electric conductivity in 1:5 soil extracts; DW, dry weight; WC%, water content percentage; Chl a, chlorophyll a; Chl b, chlorophyll b; Caro, total carotenoids; Pro, proline; GB, glycine betaine; TSS, total soluble sugars; Fru, fructose; Suc, sucrose; ions in roots and leaves, as indicated*.

ns, *, **, *** *indicate non-significant or significant at p < 0.05, p < 0.01, or p < 0.001, respectively*.

**Figure 3:**
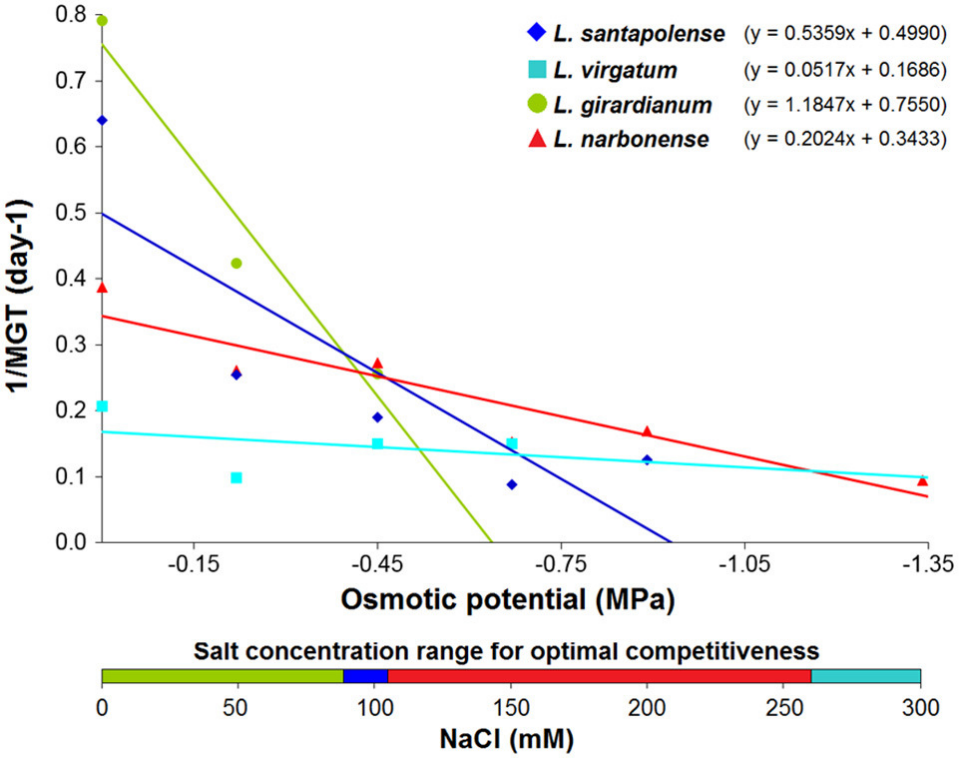
Progression of the germination velocity in relation to the osmotic potential of the germination medium, generated by the applied NaCl concentrations, in the four *Limonium* species. The bottom bar represents the salt concentration ranges corresponding to higher competitiveness for each species, determined by the intersection points of the regression lines.

Comparison of seed germination behavior of the four *Limonium* species was also addressed by calculating, from the linear regression equations shown in Figure 3, two theoretical values: base osmotic potential (Ψb) and hydrotime (θ) of each species (Table S1). Ψb values should be considered with caution, as they were obtained by extrapolation. Nevertheless, these data provided information about the competitiveness of each species in relation to the osmotic potential of the germination medium (Trudgill et al., 2005; Zhang et al., 2016). According to these calculations, the most competitive species at higher salt concentrations would be *L. narbonense* (above 103.6 mM) and *L. virgatum* (above 259.2 mM); the latter is the species showing lowest Ψb and highest θ values (Table S1). When seeds were exposed to low salt concentrations (below 89.2 mM), the most competitive species is *L. girardianum*, which showed the highest germination velocity under these conditions. Consequently, attending to their germination competitiveness against rising salt concentrations, the investigated *Limonium* species could be ordered as *L. girardianum* < *L. santapolense* < *L narbonense* < *L. virgatum* (Figure 3).

**Figure 4:**
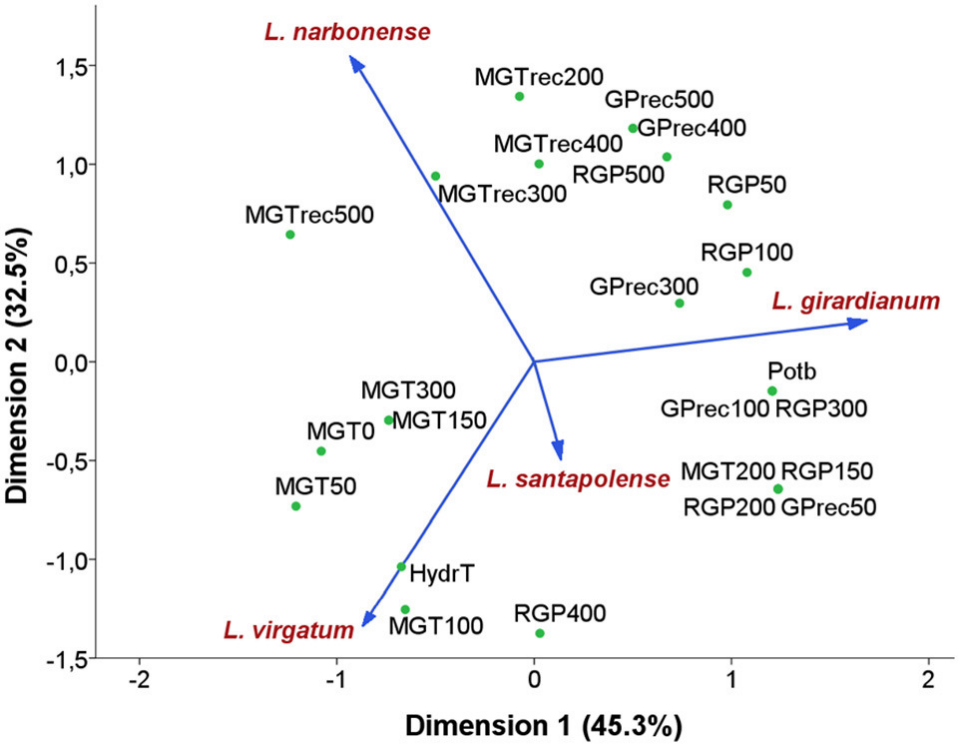
Ordination plot of the four *Limonium* species using Nonlinear Principal Components Analysis (NLPCA). Species are indicated by vectors. Abbreviations of the indicated variables: RGP, reduction of germination percentage; MGT, mean germination time; GPrec, germination percentage in recovery; MGTrec, mean germination time in recovery; Potb, base osmotic potential; HydrT, hydrotime. Numbers in acronyms indicate the tested NaCl concentrations.

All previous data were analyzed applying an NLPCA (nonlinear principal component analysis), to establish the variables with the greatest influence on the response to salt stress of the selected *Limonium* species, at the seed germination stage (Figure 4). The NLPCA results indicated that all variables considered could be represented by two new variables or dimensions, which together explained 77.8% of the total variance—45.3% corresponding to the first dimension and 32.5% to the second one. The Cronbach's alpha values indicated high internal consistency; they were close to one for both components, 0.952 and 0.917, respectively, with a total of 0.989.

The graphic representation of the results depicts the distribution of the four species according to their germination response (Figure 4). The first axis correlated positively with the reduction of germination percentages (RGP) and the decrease of germination velocity (increase in MGT) at high salt concentrations. This axis corresponds therefore to seed germination capacity, and allowed separating the most salt tolerant and widespread species, *L. virgatum* and *L. narbonense*, from the less tolerant *L. girardianum* and *L. santapolense*; the latter taxon, with a more restricted distribution area, exhibited an intermediate behavior. The second axis correlated mainly with seeds response to salt priming and discriminated between the two most tolerant and competitive species under high salt conditions (*L. virgatum* and *L*. *narbonense*). *L. virgatum* showed less tolerance and lower germination rates at low salinity than *L*. *narbonense*, but a higher recovery capacity, as it is the species with a more rapid germination (lower MGT) after seed exposure to salt. Therefore, germination velocity, both in the initial germination assays in the presence of NaCl and in the recovery tests, is the most important discriminating factor.

### Electric conductivity in the soil and in pot substrates

Soil samples from “Clot de Galvany” (where *L. santapolense* seeds were collected) had significantly higher electrical conductivity (EC_1:5_ = 8.9 ± 0.5 dS m^−1^) than those from “La Albufera” Natural Park, for which average EC_1:5_ values in the collection sites of the other three species varied from 4.3 to 6.1 dS m^−1^ (although these differences were statistically non-significant). It seems, therefore, that *L. santapolense* is adapted to higher salinity in its natural environment.

Regarding the greenhouse experiments, after 1 month of salt treatments EC_1:5_ of pot substrates increased in parallel with increasing NaCl concentrations for all four species, as expected, reaching values of around 17 dS m^−1^ in the pots watered with 400 mM NaCl, and of 30 dS m^−1^, approximately, in the presence of 800 mM. Relatively small differences, in most cases not significant, were observed when applying the same salt treatment to the different *Limonium* taxa (Table S2). Although soil salinity in the field and in the pots cannot be directly compared quantitatively, it is clear that plants watered in the greenhouse with NaCl concentrations above 200 mM have been subjected to much stronger salt stress conditions than those growing in nature.

### Effects of salt stress on plant growth and photosynthetic pigments levels

Salt treatments generally lead to a concentration-dependent inhibition of growth, which is best quantified by the relative reduction of the DW of the plant aerial part, as compared to the non-stressed control. The four studied *Limonium* species, however, showed an extremely high tolerance to salinity, so that reduction of biomass accumulation was statistically significant only in the presence of the highest NaCl concentrations tested. In *L. santapolense, L. girardianum*, and *L. narbonense*, after 1-month salt treatments, a significant reduction of leaf DW (between 20 and 30% of the corresponding controls) was only observed in the plants watered with 800 mM NaCl. In *L. virgatum*, not only there was no inhibition of growth in the presence of salt, but a significant increase of DW (about 1.5-fold higher than in the control) was observed in plants watered with 400 mM NaCl; that is, in this species relatively high salt concentrations stimulate vegetative growth (Table 3A). Regarding reduction of leaf surface area (LA), a significant decrease in average values, ranging from 30 to 40% of the controls, was detected in the presence of 800 mM NaCl in all *Limonium* taxa, except for *L. santapolense*; this latter taxon showed a mere, non-significant reduction of about 11% in average LA under the same conditions (Table 3B). Therefore, according to these parameters, *L. virgatum* and *L. santapolense* could be considered as (slightly) more salt tolerant than the other two *Limonium* species, because of their relatively lower salt-induced growth inhibition.

**Table 3:** Salt stress effects on vegetative growth in the four studied *Limonium* species.

**Parameter**	**NaCl treatments (mM)**	**Species**			
		***L. santapolense***	***L. virgatum***	***L. girardianum***	***L. narbonense***
**(A)**					
Leaf DW (% of control)	0	100.00 ± 12.52bA	100.00 ± 19.99aA	100.00 ± 16.98bcA	100.00 ± 10.88bA
	200	85.50 ± 10.39abA	94.62 ± 15.64aAB	118.26 ± 18.95cB	98.17 ± 8.65bAB
	400	110.34 ± 13.67bA	152.61 ± 28.83bB	94.21 ± 21.14abA	97.76 ± 10.81bA
	800	78.99 ± 8.53aA	99.51 ± 14.35aB	70.41 ± 11.02aA	77.11 ± 15.08aA
**(B)**					
Leaf area (% of control)	0	100.00 ± 7.28abA	100.00 ± 4.47bA	100.00 ± 5.62bA	100.00 ± 7.52bA
	200	107.03 ± 9.25abcAB	102.14 ± 5.54bA	110.94 ± 6.23bAB	121.02 ± 9.85cB
	400	121.83 ± 8.52cC	98.75 ± 5.15bB	74.72 ± 7.15aA	97.57 ± 6.96bB
	800	88.77 ± 7.08aC	60.36 ± 3.30aA	69.12 ± 8.71aB	64.53 ± 6.52aAB
**(C)**					
WC% in leaves	0	84.55 ± 0.26bA	87.50 ± 0.46cC	86.88 ± 0.17cBC	85.22 ± 0.97cAB
	200	86.23 ± 0.32cAB	87.97 ± 1.15cB	86.18 ± 0.59bcAB	85.60 ± 0.64cA
	400	84.46 ± 0.37bA	85.26 ± 0.51bA	84.51 ± 1.70bA	83.35 ± 0.91bA
	800	80.19 ± 0.75aA	80.39 ± 0.49aA	80.36 ± 1.19aA	80.43 ± 0.70aA
**(D)**					
WC% in roots	0	66.54 ± 2.42cA	76.03 ± 0.20aB	78.82 ± 1.48bC	80.37 ± 1.02bcC
	200	61.98 ± 2.51bA	77.81 ± 1.79aB	79.43 ± 2.44bBC	82.09 ± 0.66cC
	400	55.14 ± 3.18aA	76.62 ± 1.54aB	79.38 ± 0.57bC	80.16 ± 0.81bC
	800	59.76 ± 3.96bA	77.49 ± 1.23aC	68.84 ± 2.14aB	77.63 ± 0.49aC

***(A)** Total leaf dry weight (DW), as percentage of the mean absolute values of the non-treated controls. **(B)** Leaf surface area, as percentage of the mean absolute values of the non-treated controls. **(C)** Water content percentage (WC%) in leaves. **(D)** Water content percentage (WC%) in roots. Plants were treated for 1 month with NaCl, as indicated. Values shown are means ± SE (n = 5). Different lower case letters in each column indicate statistically significant differences between treatments for the same species, and different capital letters in each row indicate statistically significant differences between species for each treatment, according to Tukey test (α = 0.05)*.

All tested *Limonium* species showed a strong resistance to salt-induced leaf dehydration, which probably contributes to their high tolerance. In plants watered with 800 mM NaCl, leaf WC was calculated to be ca. 80% in all taxa, a slight—although statistically significant—reduction as compared to the non-stressed controls, in which WC varied between 84.5 and 87.5% (Table 3C). WC in roots was lower than in leaves and also showed small reductions as a response to high salt concentrations, except in *L. virgatum*, in which non-significant changes were detected (Table 3D).

Salt treatments caused a reduction in the leaf contents of photosynthetic pigments (chlorophylls a and b, and total carotenoids) in the four *Limonium* species, although only at elevated NaCl concentrations (Table 4). This is in agreement with the high salt tolerance of these taxa revealed by the growth inhibition experiments. In general, a significant decrease of pigments levels was only detected in the presence of 800 mM NaCl, except for Chl b in *L. virgatum* and Caro in *L. virgatum* and *L. narbonense*, which were significantly lower than in the controls also at 400 mM. It is worth mentioning the significant increase of Chl a in *L. virgatum*, in the presence of 200 and 400 mM NaCl (Table 4A), which correlated with the salt-induced growth stimulation observed in this species.

**Table 4:** Photosynthetic pigments levels in leaves of the four studied *Limonium* species.

**Parameter**	**NaCl treatments (mM)**	**Species**			
		***L. santapolense***	***L. virgatum***	***L. girardianum***	***L. narbonense***
**(A)**					
Chl a (mg g^−1^ DW)	0	2.37 ± 0.17bB	1.74 ± 0.18bA	2.63 ± 0.12bB	1.97 ± 0.19bA
	200	2.38 ± 0.13bB	2.20 ± 0.06cA	2.56 ± 0.22bAB	2.14 ± 0.10bA
	400	2.13 ± 0.29abA	2.01 ± 0.12cA	2.30 ± 0.22bA	2.20 ± 0.08bA
	800	1.88 ± 0.16aB	1.46 ± 0.07aA	1.83 ± 0.05aB	1.92 ± 0.03aB
**(B)**					
Chl b (mg g^−1^ DW)	0	1.11 ± 0.01bB	1.10 ± 0.06bAB	1.07 ± 0.10bAB	1.00 ± 0.14bA
	200	1.16 ± 0.12bA	1.20 ± 0.11bA	1.03 ± 0.08bA	1.04 ± 0.10bA
	400	1.08 ± 0.06bB	0.88 ± 0.07aA	1.00 ± 0.14bAB	0.94 ± 0.11bAB
	800	0.88 ± 0.06aB	0.80 ± 0.11aAB	0.71 ± 0.05aA	0.71 ± 0.07aA
**(C)**					
Caro (mg g^−1^ DW)	0	1.19 ± 0.07bAB	1.36 ± 0.11bB	0.97 ± 0.08bA	1.30 ± 0.21cB
	200	1.32 ± 0.14bB	1.25 ± 0.10bB	0.92 ± 0.12bA	1.12 ± 0.11cAB
	400	1.26 ± 0.13bC	0.88 ± 0.14aA	1.06 ± 0.08bBC	0.95 ± 0.07bAB
	800	1.03 ± 0.05aC	0.89 ± 0.12aBC	0.68 ± 0.05aA	0.79 ± 0.06aB

***(A)** Chlorophyll a (Chla), **(B)** chlorophyll b (Chl b), and **(C)** total carotenoids (Caro) contents, expressed in mg g^-1^ DW, were determined in plants treated for 1 month with NaCl, at the indicated concentrations. Values shown are means ± SE (n = 5). Different lower case letters in each column indicate statistically significant differences between treatments for the same species, and different capital letters in each row indicate statistically significant differences between species for each treatment, according to Tukey test (α = 0.05)*.

Two-way ANOVA revealed significant effects of the variable “species” on all analyzed parameters related to plant growth and photosynthetic pigments levels, except leaf DW and Chl b. When referring to the variable “treatment,” significant effects were observed for all parameters but DW. However, the interaction “species” × “treatment” was non-significant for all measured parameters with the exception of root WC (Table 2).

### Ion accumulation in NaCl-treated *Limonium* plants

**Figure 5:**
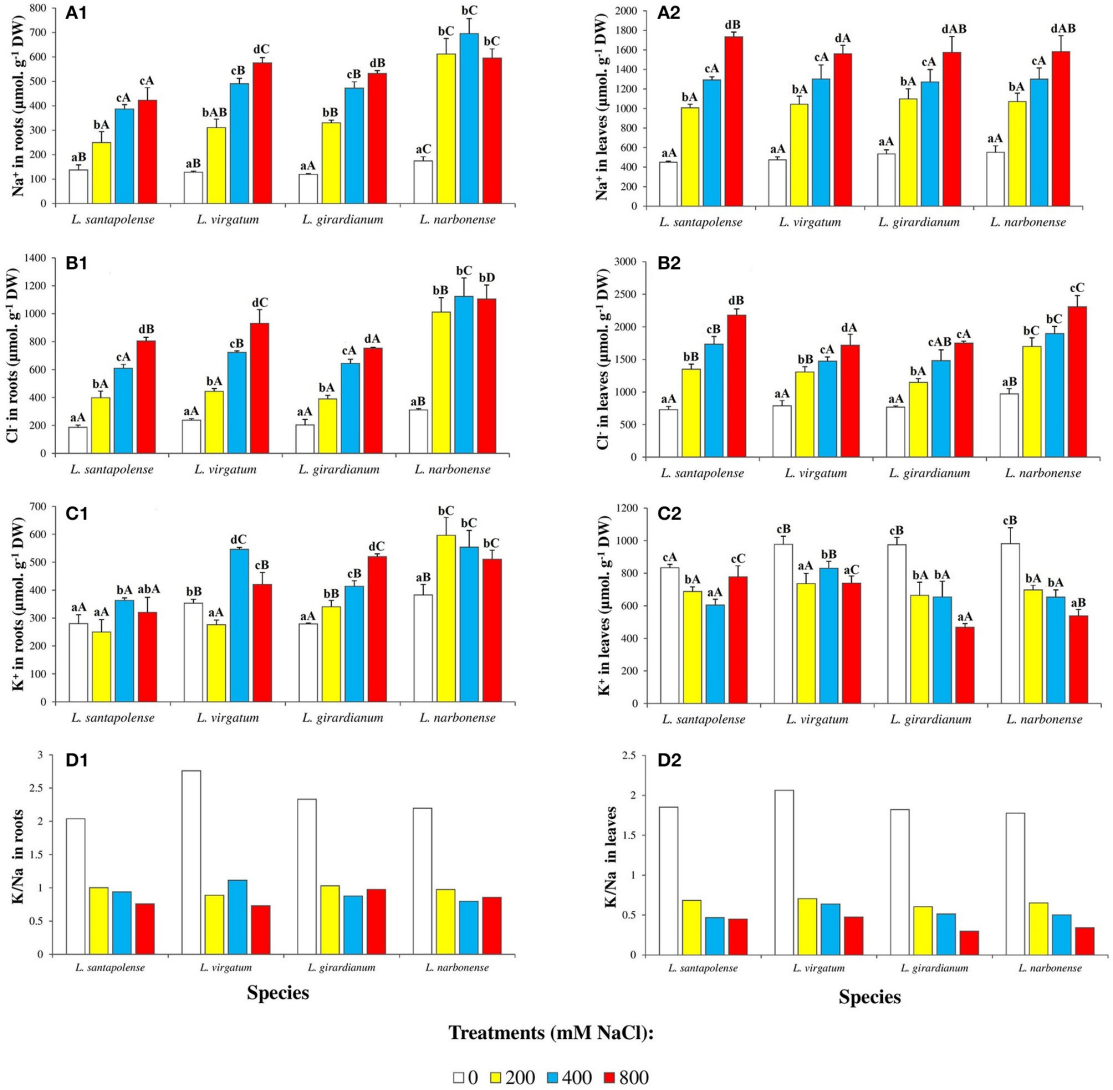
Monovalent ion concentrations in roots and leaves of the four studied *Limonium* species. Sodium (Na^+^) in roots **(A1)** and leaves **(A2)**, chloride (Cl^−^) in roots **(B1)** and leaves **(B2)**, potassium (K^+^) in roots **(C1)** and leaves **(C2)**, and potassium over sodium ratio (K^+^/Na^+^) in roots **(D1)** and leaves **(D2)** of *Limonium* plants, after 1 month of salt treatments at the indicated NaCl concentrations. The values shown are means with SE (*n* = 5). Different lowercase letters above the bars indicate significant differences between treatments for each species, and different capital letters indicate significant differences between species for each treatment, according to Tukey's test (α = 0.05).

Sodium and chloride levels increased in the roots and leaves of *Limonium* plants, in parallel to increasing external salinity, in the four investigated species. Yet absolute contents of these toxic ions were higher in leaves (between 2- and 4-fold) than in roots, for a given NaCl concentration (Figures 5A,B). Salt-dependent Na^+^ accumulation in roots was lower in *L. santapolense*, reaching ca. 400 μmol g^−1^ DW in the presence of 800 mM NaCl, as compared to around 500 μmol g^−1^ DW measured in *L. irgatum, L. girardianum*, and *L. narbonense*. This latter species differed from the other three in that maximum Na^+^ levels were also measured in roots of plants treated with 200 or 400 mM NaCl (Figure 5A1). These differences were not observed in the leaves, where the patterns of Na^+^ accumulation, as well as the concentrations measured at each salinity level in the pots (1,560–1,730 μmol g^−1^ DW at 800 mM NaCl), were similar in all species under study (Figure 5A2). Accordingly, two-way ANOVA did not detect significant differences in the variable “species,” regarding Na^+^ contents in leaves (Table 2). Salt-dependent Cl^−^ accumulation in roots followed the same pattern than that of Na^+^, albeit with higher absolute values (750–1,100 μmol g^−1^ DW, in the presence of 800 mM NaCl; Figure 5B1). It was similar also in leaves, except that some differences were observed between the *Limonium* taxa, as Cl^−^ contents measured in *L. virgatum* and *L. girardianum* (1,718 and 1,750 μmol g^−1^ DW, respectively) were significantly lower than in *L. santapolense* and *L. narbonense* (2,180 and 2,300 μmol g^−1^ DW, respectively; Figure 5B2). These differences were also significant according to the results of two-way ANOVA (Table 2).

Potassium contents in roots increased in response to salt stress in the four *Limonium* taxa, although with different accumulation patterns (Figure 5C1). In *L. virgatum, L. girardianum*, and *L. narbonense*, maximum K^+^ levels were not significantly different (520–600 μmol g^−1^ DW), and were higher than in *L. santapolense*. In this latter species and in *L. virgatum*, significant increases over the controls were observed at 400 and 800 mM external NaCl, while in *L. girardianum* K^+^ accumulation was progressive, and detected starting at 200 mM salt concentration. Similar to what was observed for Na^+^ and Cl^−^ (Figures 5A1,B1), K^+^ reached maximum levels in *L. narbonense* roots already in the presence of 200 mM NaCl (Figure 5C1). Opposite to roots, K^+^ levels in the leaves decreased in all four *Limonium* species with increasing salinity (Figure 5C2). In *L. santapolense* and *L. virgatum* the reduction in K^+^ contents with respect to the non-stressed plants was significant in the presence of 200–400 mM NaCl, but did not decrease more, or even increased slightly at higher salt concentrations; *L. girardianum* and *L. narbonense* showed a stronger and progressive K^+^ reduction, in parallel to increasing NaCl in the watering solution, down to about 50% of the corresponding controls (Figure 5C2). Differences in K^+^ levels, between species and treatments, in both roots and leaves, were significant, according to two-way ANOVA (Table 2).

Salt-induced changes in K^+^/Na^+^ ratios in roots and leaves of the four studied species are shown in Figures 5D1,D2, respectively. The general qualitative patterns of variation were similar in both organs and in all *Limonium* taxa: a clear reduction in salt-treated plants with respect to the controls, with smaller differences in K^+^/Na^+^ ratios for the different salt treatments. For example, in leaves of non-stressed plants K^+^/Na^+^ ratios varied between 1.8 and 2.1, and were reduced to ~35% of control values in the presence of 200 mM NaCl, and down to about 20% at 800 mM (Figure 5D).

**Figure 6:**
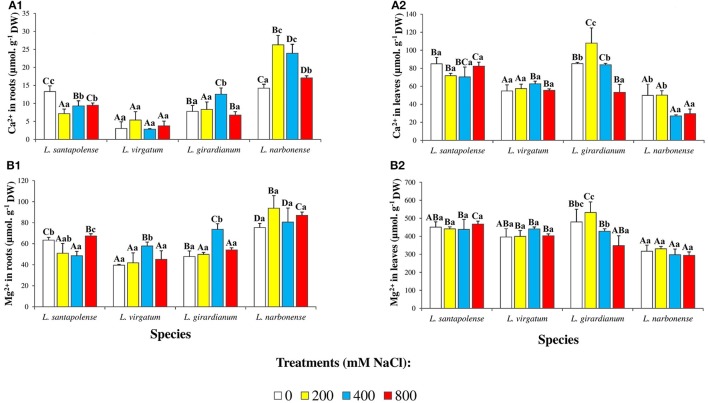
Divalent ion concentrations in roots and leaves of the four studied *Limonium* species. Calcium (Ca^2+^) in roots **(A1)** and leaves **(A2)**, and magnesium (Mg^2+^) in roots **(B1)** and leaves **(B2)** of *Limonium* plants, after 1 month of salt treatments at the indicated NaCl concentrations. The values shown are means with SE (*n* = 5). Different lowercase letters above the bars indicate significant differences between treatments for each species, and different capital letters indicate significant differences between species for each treatment, according to Tukey's test (α = 0.05).

Divalent cations (Ca^2+^, Mg^2+^) levels were also determined in roots and leaves of the *Limonium* plants (Figure 6). Apart from the fact that, in all cases, cation contents were higher in leaves than in roots, no common salt-induced variation pattern was observed in the different species. For example, Ca^2+^ concentrations were similar in roots of non-treated *L. santapolense* and *L. narbonense* plants (13–14 μmol g^−1^ DW) and decreased slightly in the former species in response to salt stress, while almost doubled in the latter. In *L. virgatum*, which had much lower Ca^2+^ levels in control roots (3 μmol g^−1^ DW), no significant changes were observed in the presence of NaCl (Figure 6A1). Mg^2+^ contents in roots of control plants varied between 40 μmol g^−1^ DW (in *L. virgatum*) and 75 μmol g^−1^ DW (in *L. narbonense*), and changes induced by salt stress were small, in most cases statistically non-significant (Figure 6B1). Concerning Ca^2+^ contents in leaves, the patterns of variation in the presence of salt were different in *L. santapolense* and *L. virgatum*—showing no significant changes—on the one hand, and in *L. girardianum* and *L. narbonense*—showing a significant decrease at high NaCl concentrations—on the other (Figure 6A2). Measured leaf Mg^2+^ concentrations were relatively high in control plants, from 317 μmol g^−1^ DW (in *L. narbonense*) to 480 μmol g^−1^ DW (in *L. girardianum*), and did not change in salt-stressed plants, except in *L. girardianum*, which showed a significant reduction, although only in the presence of 800 mM NaCl (Figure 6B2).

### Monovalent ions on the leaf surface of *Limonium* plants

**Figure 7:**
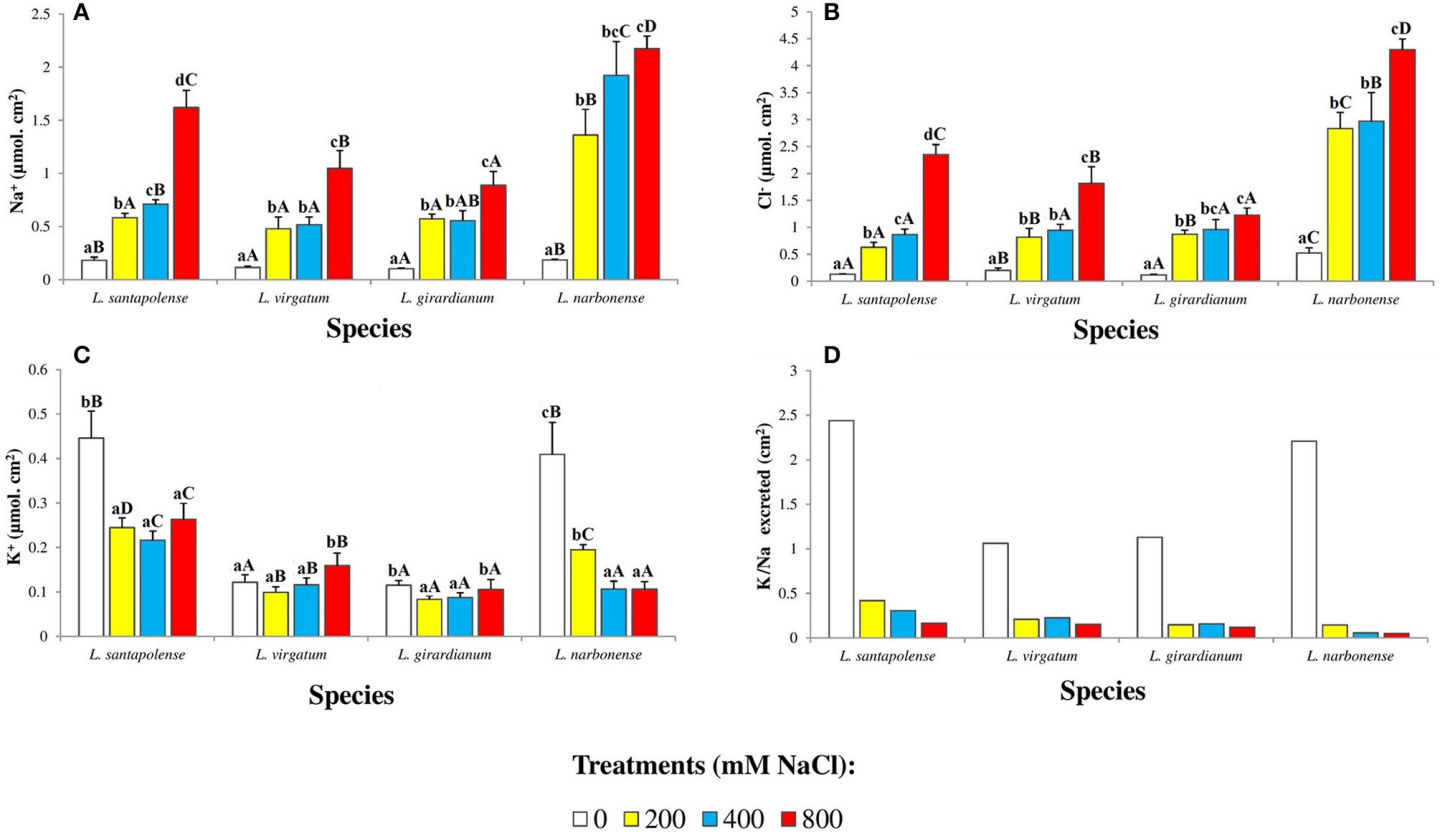
Monovalent ions accumulated on the leaf surface of the four studied *Limonium* species. Sodium (Na^+^) **(A)**, chloride (Cl^−^) **(B)**, and potassium (K^+^) **(C)** amounts, expressed in μmol per cm^2^ of leaf surface area, and potassium over sodium ratio (K^+^/Na^+^) **(D)**, after 1 month of salt treatments at the indicated NaCl concentrations. The values shown are means with SE (*n* = 5). Different lowercase letters above the bars indicate significant differences between treatments for each species, and different capital letters indicate significant differences between species for each treatment, according to Tukey's test (α = 0.05).

The amounts of monovalent ions (Na^+^, Cl^−^, and K^+^), secreted by salt glands and accumulated on the leaf surface, were determined in salt-treated *Limonium* plants (Figure 7). To standardize the results, since leaf size varied in the four investigated species, the obtained values were expressed as μmol ion per cm^2^ of leaf surface. In control plants, Na^+^ levels were low and similar in *L. virgatum* and *L. girardianum* (ca. 0.1 μmol cm^−2^), and also in *L. santapolense* and *L. narbonense* (0.18 μmol cm^−2^). Sodium on the leaf surface increased with increasing external salinity, to reach 12-fold higher levels over the control in *L. narbonense*, and about 9-fold in the other three species, in the presence of 800 mM NaCl (Figure 7A). The patterns of Cl^−^ accumulation on leaf surfaces were qualitatively similar to those of Na^+^, with quantitative differences between species: at the highest salt concentration tested, the relative increases over the controls ranged between 8- and 18-fold for the different taxa, with *L. narbonense* reaching the highest Cl^−^levels (4.3 μmol cm^−2^), and *L. girardianum* the lowest (1.2 μmol cm^−2^; Figure 7B).

Regarding K^+^ ions on the leaf surfaces, the *Limonium* species could be separated into two groups. In *L. santapolense* and *L. narbonense*, relatively high K^+^ levels (0.40–0.45 μmol cm^−2^) were measured in the controls, decreasing to 50 and 25%, respectively, in the presence of 800 mM external NaCl. In *L. virgatum* and *L. girardianum*, on the other hand, control K^+^ values were much lower (0.12 μmol cm^−2^, approximately) but did not change, or changed very little, upon salt treatment of the plants (Figure 7C). Salt-induced changes in Na^+^ and K^+^ resulted in the K^+^/Na^+^ ratios shown in Figure 7D, with higher control values in *L. narbonense* and *L. santapolense* (2.2–2.4) than in *L. virgatum* and *L. girardianum* (ca. 1), and drastic reductions in salt-treated plants of the four species.

### Salt-induced osmolyte accumulation

**Figure 8:**
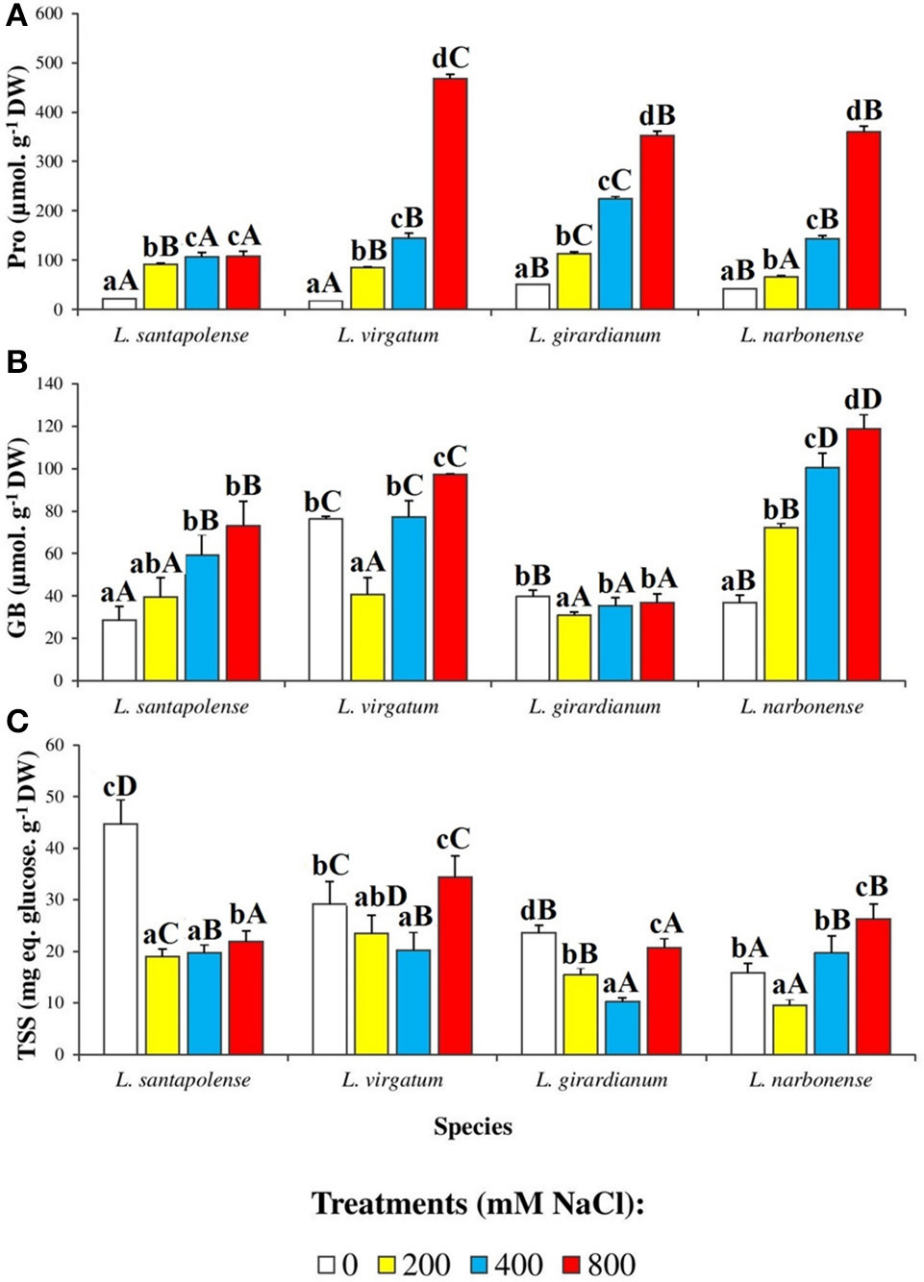
Osmolyte contents in leaves of the four studied *Limonium* species. Proline (Pro) **(A)**, glycine betaine (GB) **(B)**, and total soluble sugar (TSS) levels **(C)**, after 1 month of salt treatments at the indicated NaCl concentrations. The values shown are means with SE (*n* = 5). Different lowercase letters above the bars indicate significant differences between treatments for each species, and different capital letters indicate significant differences between species for each treatment, according to Tukey's test (α = 0.05).

The levels of the most common osmolytes in plants—proline (Pro), glycine betaine (GB) and total soluble sugars (TSS)—were determined in leaves of the investigated *Limonium* taxa, after concluding the salt stress treatments (Figure 8). Pro contents measured in control plants were more than twofold higher in *L. narbonense* and *L. girardianum* (42 and 52 μmol g^−1^ DW, respectively) than in *L. santapolense* and *L. virgatum* (about 20 μmol g^−1^ DW), and increased in all cases with increasing salinity; there were however, clear quantitative differences between species. The strongest Pro accumulation in plants treated with 800 mM NaCl was observed in *L. virgatum*, reaching almost 470 μmol g^−1^ DW (a 25-fold increase over the control). The lowest level, about 100 μmol g^−1^ DW (5-fold increase), was determined in *L. santapolense*. Intermediate changes were detected in *L. girardianum* and *L. narbonense*, with maximum Pro contents of 350–360 μmol g^−1^ DW, representing increases of 7- to 8.5-fold (Figure 8A).

Leaf GB contents also increased in parallel to salt concentrations in the stress treatments, in all *Limonium* species except *L. girardianum*, in which non-significant or very small salt-induced changes were observed. The maximum GB levels (120 μmol g^−1^ DW, 3.2-fold higher than the control) were reached in *L. narbonense* (Figure 8B). Yet these values were in all cases clearly lower than those of Pro.

**Figure 9:**
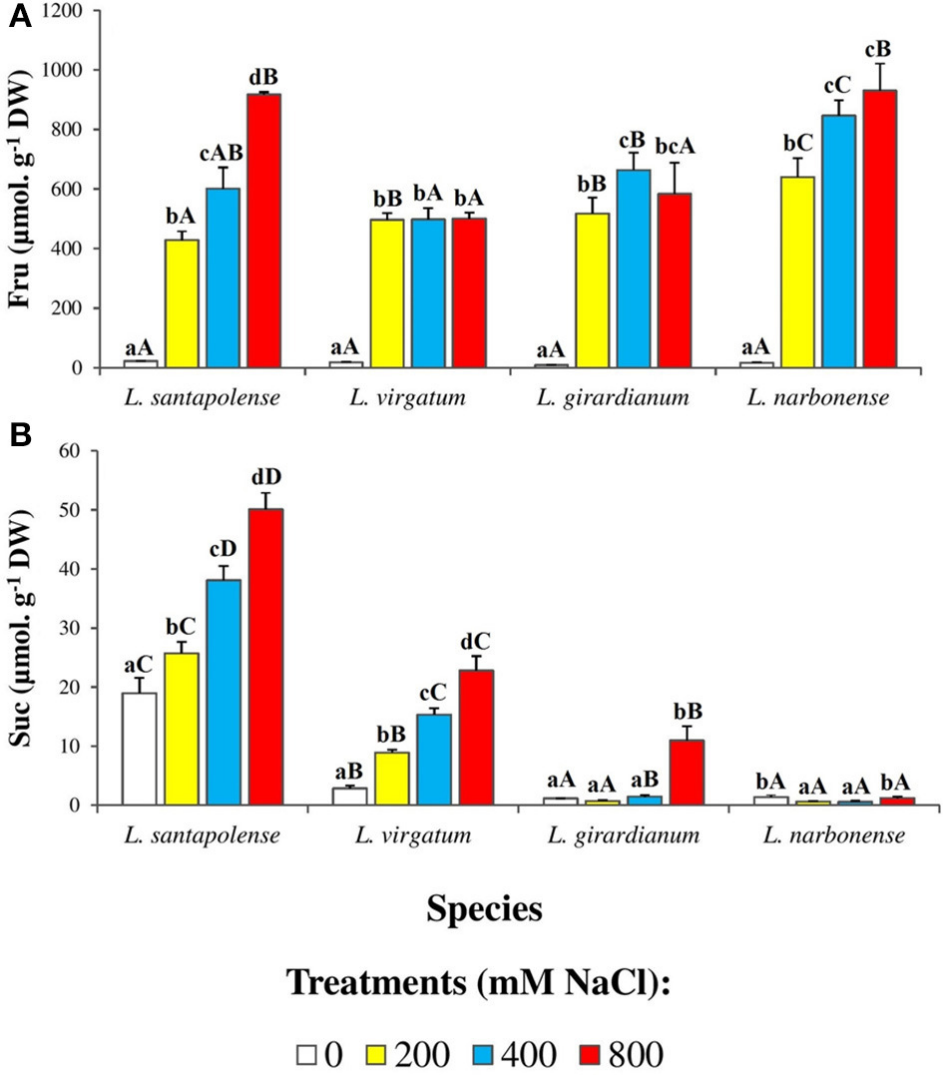
Soluble sugars contents in leaves of the four studied *Limonium* species. Fructose (Fru) **(A)** and sucrose (Suc) **(B)** levels, after 1 month of salt treatments at the indicated NaCl concentrations. The values shown are means with SE (*n* = 5). Different lowercase letters above the bars indicate significant differences between treatments for each species, and different capital letters indicate significant differences between species for each treatment, according to Tukey's test (α = 0.05).

No clear correlation of TSS contents and salt treatments could be observed, between or within the four *Limonium* taxa (Figure 8C). Therefore, carbohydrates in the leaf water soluble fraction were separated, identified and quantified by HPLC (Figure 9). Only two major peaks were detected in the chromatograms, corresponding to fructose (Fru), and sucrose (Suc). Glucose could also be measured in some samples, at very low levels, but it was below the detection limit in most extracts (data not shown). Fru concentrations increased in response to salt stress in all four *Limonium* taxa. In *L. santapolense* and *L. narbonense* Fru increased progressively, more or less in parallel with external salinity, reaching maximum concentrations (>900 μmol g^−1^ DW) at 800 mM NaCl. In *L. virgatum* and *L. girardianum* the pattern was somewhat different: Fru levels of ca. 500 μmol g^−1^ DW were measured in the presence of 200 mM external NaCl but did not increase or increased only slightly at higher salt concentrations (Figure 9A). Absolute leaf Suc contents and their changes in response to salt stress varied widely in the different species; they were very low in non-stressed *L. girardianum* and *L. narbonense* plants, increasing significantly only in the presence of 800 mM NaCl in the former species, and not at all in the latter. Control Suc levels were much higher (about 15-fold) in *L. santapolense*, and increased progressively in response to salt stress; in *L. virgatum* Suc contents also increased in the presence of NaCl, in a concentration-dependent manner, but with lower absolute values at each salt concentration (Figure 9B). In any case, even in *L. santapolense*, which accumulated the highest levels—up to 50 μmol g^−1^ DW—Suc cannot contribute significantly to osmotic adjustment since this value was about 10-fold lower than the minimum Fru concentration determined in the presence of salt in all *Limonium* species.

### Principal component analysis (PCA)

**Figure 10:**
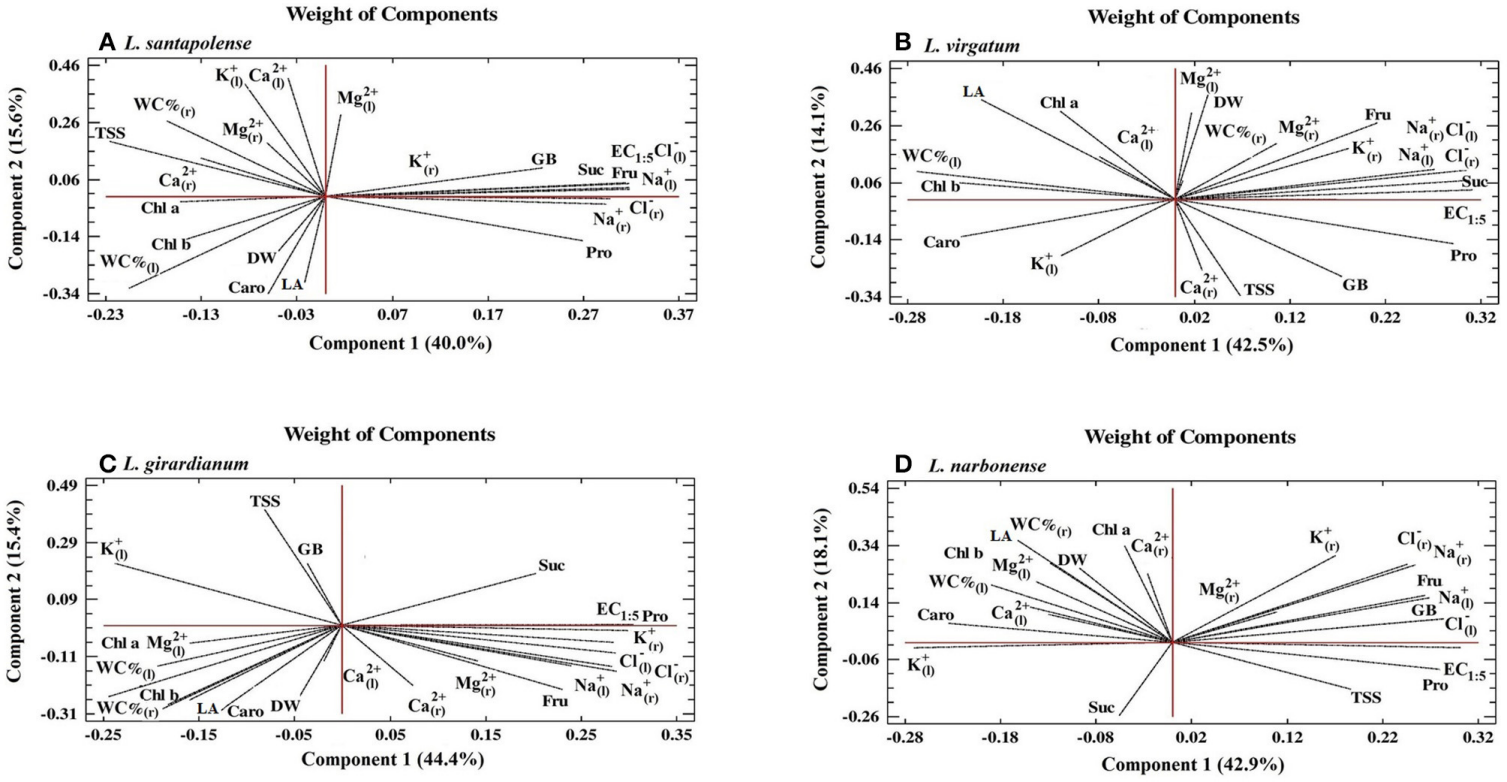
Principal Component Analyses (PCAs). Changes in all measured growth parameters and biochemical stress markers (photosynthetic pigments, ions, and osmolytes) were analyzed in salt-treated plants with respect to the control, non-stressed plants of the investigated *Limonium* species: *L. santapolense* **(A)**, *L. virgatum* **(B)**, *L. girardianum* **(C)**, and *L. narbonense* **(D)**, in correlation to EC (electrical conductivity) of the substrate, at the end of the 1-month salt treatment. DW, dry weight; LA, leaf surface area; WC%_(l)_, water content percentage in leaves; WC%_(r)_, water content percentage in roots; Chl a, chlorophyll a; Chl b, chlorophyll b; Caro, total carotenoids; Pro, proline; GB, glycine betaine; TSS, total soluble sugars; Fru, fructose; Suc, sucrose; Na^+^, sodium; K^+^, potassium; Cl^−^, chloride; Ca^2+^, calcium; Mg^2+^, magnesium; ions in roots (“r” subscript) and leaves (“l” subscript).

PCAs were carried out, independently for each species, to correlate all variables determined after the salt stress treatments. Five (in *L. girardianum*) or six (in the other three taxa) components showed Eigenvalues higher than 1. Biplots of the two main principal components are shown in Figure 10; together, they explain between 55 and 61% of the total variability, depending on the species. The first component (X-axis), explaining in all cases ≥40% of the total variability, correlated with the EC_1:5_ of the substrate; that is, with the intensity of salt stress applied to the plants. In general, growth parameters and photosynthetic pigments showed negative correlations with EC_1:5_, in agreement with the observed inhibition of growth and pigment degradation with increasing salinity. However, the angles of the vectors corresponding to dry weight (DW) and leaf surface area (LA) with the X-axis indicated weak correlations—the smaller the angle, the stronger the correlation—which can be explained by the relatively high tolerance to salt of all investigated species. In fact, for the most tolerant *L. virgatum* (Figure 10B) the correlation of DW with salinity was positive (although very weak, since the vector was much closer to the Y-axis), reflecting the lack of inhibition and even stimulation of growth observed in this species in the presence of NaCl. Some additional species-specific differences were detected for other parameters, indicating weak negative correlations with salinity, for example for Chla in *L. virgatum* (Figure 10B) and *L. narbonense* (Figure 10D), or for Caro in *L. santapolense* (Figure 10A) and *L. giradianum* (Figure 10C).

Strong positive correlations with the X-axis were detected, in all *Limonium* taxa, for Na^+^ and Cl^−^ in roots and leaves, in accordance with the observed increase in their levels in response to increasing salinity. K^+^ ions showed the same qualitative pattern in the four species: a positive correlation with EC_1:5_ in roots (where an increase in K^+^ concentration was generally observed in salt-treated plants) and a negative correlation in leaves (where raising external salt concentrations led to a reduction of K^+^ levels); however, this negative correlation was relatively weaker in the most tolerant species, *L. santapolense* and *L. virgatum*, which showed reduction of K^+^ only at low or moderate NaCl concentrations (see Figure 5C2). Divalent cations (Ca^2+^, Mg^2+^) did not show any clear pattern, neither in roots nor in leaves: positions of the corresponding vectors were variable for the different taxa and, in general, weak or no correlation with salinity could be established.

Pro and Fru, which appear to be the major functional osmolytes, responsible for osmotic adjustment under salt stress conditions, also showed strong positive correlations with salinity in the four *Limonium* taxa. Some differences between species were found regarding putative “secondary” osmolytes. A positive correlation with the X-axis was established for GB in *L. santapolense, L. narbonense* and (weaker) in *L. virgatum*, but no correlation in *L. giradianum*; positive correlations of Suc with salinity were also detected in all species except *L. narbonense* (Figure 10).

Summarizing, the joint statistic assessment by PCA of all variables determined in the salt treatments of the selected *Limonium* taxa, confirmed the conclusions of the individual experiments described in the previous sections.

## Discussion

### Selected *Limonium* species and experimental strategy

*Limonium* is an extremely interesting genus, including a large number of species that are generally adapted to saline environments—some to arid habitats or gypsum soils—with a great taxonomic and functional diversity (Erben, 1993; Brullo and Erben, 2016). In the present study, we have performed a comparative analysis of the responses to controlled salt stress treatments in four *Limonium* species, at the seed germination stage and during early vegetative growth. The selected taxa are closely related but present different geographic ranges, including two species widespread in the Mediterranean region and two endemic species, one of them a narrow local endemism. The results obtained help to explain the distribution in nature of the investigated taxa, based on their germination behavior in the presence of salt. Our work also allowed to define general responses to stress—dependent on regulation of ion transport and accumulation of specific osmolytes—which appear to contribute most significantly to salt tolerance. To our knowledge, this is the first systematic study of this kind in *Limonium*. Most previously published reports using similar approaches had a much more limited scope, focusing on a single *Limonium* species at a particular developmental stage, and generally characterizing a narrower range of salt-induced responses (Zia and Khan, 2004; Tabot and Adams, 2014; Souid et al., 2016). Moreover, there are very few studies focused on comparative analyses of stress responses in congener species with different scales of geographic distribution; for example, those carried out in *Artemisia* (Ishikawa and Kachi, 2000) or *Gypsophila* (Soriano et al., 2014).

The strategy of comparing the responses to salt stress of genetically related taxa adapted to different habitats—and therefore with expected differences in salinity tolerance—represents a useful tool for deciphering salt tolerance mechanisms. The rationale behind such comparative studies is that, if a specific response to salt stress contributes significantly to tolerance, it should be more efficiently activated in the more tolerant taxa (Pang et al., 2010; Kumari et al., 2015). We have successfully used this approach in previous studies in other genera of wild plants, such as *Juncus* (Al Hassan et al., 2016b, 2017), *Plantago* (Al Hassan et al., 2016c), or *Inula* (Al Hassan et al., 2016a).

### Seed germination behavior: the main factor determining geographic distribution patterns

The studied *Limonium* species showed different levels of salt tolerance at the seed germination stage. The two endemic taxa, *L. girardianum* and *L. santapolense*, were shown to be the most sensitive, as significant reductions in germination percentages were observed in the presence of only 50 and 100 mM NaCl, respectively. *Limonium narbonense* appeared to be the most tolerant, since no (or very slight) inhibition of germination was detected up to 200 mM NaCl. Salt concentrations inhibitory for seed germination have also been determined in other *Limonium* species. Studies on several Mediterranean endemics revealed a complete inhibition of germination by salt concentrations higher than 200 mM NaCl, for example in *L. cossonianum* (Giménez Luque et al., 2013), *L. tabernense* (Delgado Fernández et al., 2016), or *L. insigne* (Delgado Fernández et al., 2015). In two endemics from Turkey, *L. iconicum* and *L. lilacinum*, germination was reduced to <20% of the control at 300 mM salt (Yildiz et al., 2008). Some *Limonium* taxa are more tolerant to salt during the seed germination phase, as it is the case for *L. stocksii*, a species from India and Pakistan in which germination was optimal (and close to 100%) at or below 200 mM NaCl; germination percentages were reduced only by 40% at 300 mM, and some seeds germinated even in the presence of 400 mM NaCl (Zia and Khan, 2004).

Besides the range of salt concentrations that directly inhibits germination, the response of seeds to a previous exposure to salt is relevant for the biology and ecology of halophytes. “Recovery” of germination is the term used to refer to the ability of seeds that have been maintained under high salinity conditions to germinate when transferred to fresh water (Ungar, 1991). For the four *Limonium* species, all seeds that did not germinate in the initial tests recovered full germination capacity after the salt treatments. This response was especially relevant in *L. virgatum*, which showed a salt “priming” effect above 200 mM NaCl, germinating in water at percentages significantly higher than the non-treated controls. This seed priming effect may stimulate the first defense mechanisms against salinity stress, such as osmotic adjustment and activation of antioxidant defense systems (Ibrahim, 2016). Recovery of germination has been described in other *Limonium* species (e.g., Zia and Khan, 2004), as in many other salt tolerant species showing optimal germination in fresh water or at low salinities (Ungar, 2001; Gul et al., 2013). In fact, it is a well-known ability of halophyte seeds to survive hypersaline conditions in the soil, often for prolonged periods of time. In their natural habitats, non-germinated *Limonium* seeds should be able to withstand very high soil salinity and germinate when abundant rainfall (typical in autumn and spring in the Mediterranean climate) reduces salt concentration in the surface soil layers.

The seed germination behavior of the investigated *Limonium* species established in the present study can explain their different geographic distribution patterns. The two taxa that are widely spread in salt marshes throughout the Mediterranean region, *L. narbonense* and *L. virgatum*, are the most salt-tolerant, and the most competitive at higher salt concentrations. Therefore, they are able to colonize habitats with higher soil salinity, where they avoid competition with less tolerant species; they can also better adapt to changing environmental conditions, thus occupying larger geographical areas. Conversely, the endemic *L. santapolense* and *L. girardianum* are less salt-tolerant at the seed germination stage, and more competitive only at lower salt concentrations, and therefore are restricted to smaller areas. These two different behavioral strategies have already been evidenced in two *Gypsophila* species, and have been correlated as well with the relative extension of their geographical ranges (Soriano et al., 2014).

### Effect of salt stress on plant growth

Our results indicate that seed germination is the developmental phase most sensitive to salinity in the *Limonium* species selected for this study, as it has been established for most plants, glycophytes and halophytes alike. Once the bottleneck of germination is overcome, in general salt tolerance increases progressively with the age of the plants (Vicente et al., 2004), and it is best assessed by measuring salt-induced inhibition of vegetative growth. Reduction of growth under stress is a general adaptive feature of plants, allowing survival in hostile environments by re-directing cell resources (energy and metabolic precursors) from primary metabolism and biomass accumulation toward the activation of specific defense mechanisms (Zhu, 2001). Somewhat surprisingly, young plants of the four *Limonium* species appeared to be extremely salt tolerant, showing no apparent damage after 1-month treatments with high salt concentrations. Only in the presence of 800 mM NaCl, small (but statistically significant) reductions on growth parameters were observed. Moreover, the four taxa showed a high resistance to salt-induced leaf dehydration and a limited degradation of photosynthetic pigments, both common deleterious effects of salt stress, which were again observed only at the highest salinity tested. These results allowed to classify *L. virgatum*, followed by *L. santapolense* as (slightly) more tolerant than *L. narbonense* and *L. giradianum*. Therefore, the relative resistance to salt stress of the *Limonium* species during early vegetative growth differed from that established for germinating seeds. Under our experimental conditions, *L. virgatum* even showed a significant *stimulation* of growth (an increase of DW) at 400 mM NaCl. This behavior is common to many highly tolerant dicotyledonous halophytes, which show optimal growth at low or moderate salinities, and only salt levels above a higher, species-specific concentration threshold inhibit their growth (Flowers et al., 1986; Redondo-Gómez et al., 2006).

### Salt tolerance mechanisms based on ion transport and accumulation

One of the main differences in the general responses to salinity of monocotyledonous and dicotyledonous halophytes refers to the regulation of ionic transport and ion homeostasis. Monocot halophytes—as well as glycophytes—cope with high salinity mostly by limiting the transport of toxic ions (Na^+^ and Cl^−^) to the aerial part of the plants. Dicotyledonous salt-tolerant species, on the contrary, accumulate these ions in the leaves, where they are maintained at low cytosolic concentrations by compartmentalization in vacuoles (Flowers and Colmer, 2008; Munns and Tester, 2008). The results of our experiments are in full agreement with this latter “dicot model”: Na^+^ and Cl^−^ concentrations increased with external salinity, in a concentration-dependent manner, both in roots and leaves, and following almost identical patterns in the four investigated *Limonium* species; however, for the two ions, absolute values were considerably higher in leaves than in roots. Transport of Na^+^ and Cl^−^ from roots to leaves has also been reported in other species of *Limonium* and in many other genera (e.g., Alarcón et al., 1999; Wang et al., 2004; Al Hassan et al., 2016b), and can be considered as a strategy to maintain plant growth under salt stress conditions, using these inorganic solutes as “cheap” osmotica (Flowers et al., 1986).

Accumulation of Na^+^ in plants is generally associated with a decrease of K^+^ levels. The two cations compete for the same binding sites and Na^+^ interferes with K^+^ uptake into the cell by using its physiological membrane transport proteins. In addition, Na^+^ uptake produces a depolarization of the plasma membrane causing the activation of outward rectifying K^+^ channels and, therefore, the loss of cellular K^+^ (Flowers et al., 1977; Greenway and Munns, 1980). Considering the salt-induced changes in K^+^ concentrations in roots, and contrary to what was expected, we observed a general increase in response to increasing salinity, in the four *Limonium* species (relatively smaller in *L. santapolense*); this suggests the activation of specific K^+^ transporters, to partially compensate Na^+^ uptake. In leaves, absolute K^+^ contents were higher than in roots, and decreased in salt-treated plants, compared to the corresponding controls. It is important to point out that reduction of K^+^ was progressive in the relatively less tolerant *L. narbonense* and *L. giradianum*, with the lowest values measured in the presence of the highest salt concentration tested. In *L. virgatum* and *L. santapolense*, however, K^+^ levels decreased significantly at 200 mM NaCl, but were maintained or even augmented slightly at higher salinities. These results could be explained assuming that K^+^ transport to the leaves is activated at high external salt concentrations, which would represent a response relevant for salt tolerance in *Limonium*, since it appears to be activated specifically in the relatively more tolerant species. A similar response has been observed in our previous studies on the genus *Plantago*, both in the field (Gil et al., 2014) and in controlled salt treatments in the greenhouse (Al Hassan et al., 2016b). Further studies will be required in both species to confirm this hypothesis, by the identification and characterization of the transport proteins involved, which are as yet unknown.

In *Limonium*, a model example of recretohalophytes, toxic ions are not only sequestered in vacuoles but also secreted to the outside through salt glands; this process should also contribute to their salt tolerance. Indeed, the amounts of Na^+^ and Cl^−^ accumulated on the leaf surface increased in parallel with increasing external salinity, in the four *Limonium* taxa. Yet the simple methodology used (see Section Material and Methods), did not allow to determine actual secretion rates or establish the relative activity of salt glands in the tested species. In a comparative study on a wild *Limonium* and a cultivated hybrid, Morales et al. (2001) reported higher excretion rates of Na^+^ and Cl^−^ in the first one, which is the most tolerant. K^+^ was also eliminated by the salt glands, although the amounts of K^+^ accumulated on the leaves under high external salinity were only a fraction of those of Na^+^ (or Cl^−^), between 5 and 15%, depending on the species. This was to be expected since salt glands are generally highly selective for Na^+^ and Cl^−^ (Ramati et al., 1976; Ramadan, 1998). The main function of salt glands is secretion of excess toxic ions and the specificity of secretion mechanisms is an important feature, as random or non-selective excretion could disturb the nutritional balance of the plant (Waisel, 1972).

### Osmolyte accumulation and salt tolerance in *Limonium*

Assuming that the largest fractions of Na^+^ and Cl^−^ in the leaves are sequestered in the vacuole to avoid their toxic effects in the cytoplasm, the synthesis and accumulation of organic osmolytes are required to maintain cellular osmotic balance. In the selected *Limonium* species, this function seems to be fulfilled mainly by Fru and Pro, two common plant osmolytes. The concentration of both compounds increases in response to salt stress, reaching levels high enough to have a significant osmotic effect—apart from the potential additional functions of these compounds in the mechanisms of stress tolerance. Despite some quantitative differences in the accumulation patterns observed among the four *Limonium* species, considering the two osmolytes together, the added concentration values measured at the highest external salinity were similar in the four taxa, around 1 mmol g^−1^ DW.

Two additional compounds, GB and Suc, can be considered as “secondary” osmolytes as they also accumulate in the leaves of some of the investigated species in response to increasing salt concentrations, although reaching lower concentrations than Fru or Pro. GB could have a minor contribution to osmotic adjustment in all *Limonium* taxa, with the exception of *L. giradianum*, while salt-induced Suc accumulation has been detected in *L. santapolense* and, to a lesser extent, in *L. virgatum*. Leaf GB contents reported here are higher or similar to those measured in other *Limonium* taxa (Hanson et al., 1991), but much lower than in real GB-accumulating species (Khan et al., 2000; Tipirdamaz et al., 2006; Gil et al., 2014; Pardo-Domènech et al., 2016). Suc, on the other hand, is present at much higher levels in other *Limonium* species, for example in *L. latifolium* (Gagneul et al., 2007).

Usually, for a given species, one specific compound represents the major osmolyte, responsible for maintenance of cellular osmotic balance under stress conditions. Yet the concomitant synthesis of different compatible solutes has been previously observed in different halophytes (e.g., Briens and Larher, 1982; Tipirdamaz et al., 2006). This is also the case in our *Limonium* taxa, in which Fru and Pro have similar relevance, albeit with some species-specific quantitative differences. It is also to be expected that related species, such as those belonging to the same genus, accumulate the same osmolyte(s) as a mechanism of defense against salt stress. Apparently, this cannot be applied to *Limonium* since diverse osmolytes have been detected in different species of this genus. They include proline, quaternary ammonium compounds like β-alanine betaine, choline-0-sulfate and glycine betaine, and different soluble sugars (Fru, Suc, glucose) and polyalcohols (e.g., inositol isomers and derivatives) (Hanson et al., 1991; Rhodes and Hanson, 1993; Morales et al., 2001; Tipirdamaz et al., 2006; Gagneul et al., 2007; Liu and Grieve, 2009; Furtana et al., 2013; Tabot and Adams, 2014).

### Ecological and biodiversity implications

This study has also interest from an ecological point of view. The investigated *Limonium* taxa are present in Mediterranean littoral salt marshes, very rich and highly valuable ecosystems in risk of degradation—or already degraded—by human activities, which are also particularly sensitive to the foreseeable effects of climate change. Moreover, two of these taxa are threatened endemic species, represented by scarce populations and thus more vulnerable to changes in their natural habitats. These characteristics make them attractive candidates to be included in salt marsh conservation and regeneration programs. Knowledge on their responses to salt stress and their mechanisms of salt tolerance, derived from this work, will contribute to a better design and more efficient implementation of those programs. For example, the *Limonium* species included in this study will easily adapt to the environmental conditions of the regenerated salt marsh, even if soil salinity increases as a consequence of climate change, since they are able to withstand salt concentrations much higher than those normally encounter in their natural habitats. On the other hand, introduction of new plants for reinforcement of existing populations, for recovery of lost ones, or in newly regenerated salt marshes, can be improved using salt pre-treated seeds, which germinate better and faster. This has also economic implications, as a higher efficiency will obviously reduce management costs.

## Conclusions

The germination behavior of the investigated *Limonium* taxa appears to be responsible for their different chorology: the species with a wide distribution range, *L. narbonense* and *L. virgatum*, are the most salt-tolerant and the most competitive at higher salinities, whereas the endemic *L. santapolense* and *L. girardianum*, restricted to smaller areas, are more sensitive to salt stress and showed higher competitiveness only at lower salinities.

Seed germination is the most salt-sensitive phase of the life cycle; once it was overcome, all four *Limonium* taxa proved to be extremely tolerant to salt stress during early vegetative development, as shown by a small reduction of growth, limited degradation of photosynthetic pigments and a strong resistance to salt-induced leaf dehydration, even at high external salinities. Nonetheless, *L. virgatum* and *L. santapolense* were identified as (slightly) more tolerant than *L. girardianum* or *L. narbonense*—their relative tolerance, therefore differing from that established in the germination phase. Responses to salt stress common to all *Limonium* species include the efficient transport of Na^+^ and Cl^−^ to the leaves, where they are compartmentalized in vacuoles and partly eliminated by secretion through salt glands, and the accumulation of fructose and proline, as the main physiological osmolytes responsible for cellular osmotic adjustment. A relevant mechanism of salt tolerance is based in the apparent activation of K^+^ transport from the roots to the leaves in the presence of high salt concentrations, which has been observed in the more tolerant *L. virgatum* and *L. santapolense*.

## Author contributions

MA performed biochemical assays and collaborated on the analysis of the data and the preparation of figures and tables. EE and PS carried out all seed germination-related work, including data analysis and artwork preparation. ML and JB performed the HPLC experiments for sugar fractionation, identification, and quantification. MB was responsible for the greenhouse work and, together with OV, participated in the design, general organization and supervision of the work, and in the interpretation of the results. All authors collaborated in the preparation of the manuscript, and have read and approved its final version, prepared by OV.

### Conflict of interest statement

The authors declare that the research was conducted in the absence of any commercial or financial relationships that could be construed as a potential conflict of interest.
