# Supplementary material for: Unraveling Salt Tolerance Mechanisms in Halophytes: A Comparative Study on Four Mediterranean Limonium Species with Different Geographic Distribution Patterns
Source: Front Plant Sci. 2017 Aug 17;8:1438. doi: 10.3389/fpls.2017.01438 (PMC5562691; doi:10.3389/fpls.2017.01438)
Supplement: Supplementary file 1 [file Table1.DOCX]

**Tab. S1**. Base potential (Ψ_b_) and hydrotime (Ɵ) for the four studied *Limonium* species.

|  | Ψb (MPa) | Ɵ (MPa d) |
| --- | --- | --- |
| *L. santapolense* | -0.9 | 1.9 |
| *L. virgatum* | -3,3 | 19.3 |
| *L. girardianum* | -0.6 | 0.8 |
| *L. narbonense* | -1,7 | 4,9 |
