# Supplementary material for: Unraveling Salt Tolerance Mechanisms in Halophytes: A Comparative Study on Four Mediterranean Limonium Species with Different Geographic Distribution Patterns
Source: Front Plant Sci. 2017 Aug 17;8:1438. doi: 10.3389/fpls.2017.01438 (PMC5562691; doi:10.3389/fpls.2017.01438)
Supplement: Supplementary file 2 [file Table2.DOCX]

**Tab. S2. Electric conductivity in 1-to-5 soil extracts (EC_1:5_**) in pot substrate samples after one month of salt treatments with the indicated NaCl concentrations, expressed in dS m^-1^. Values shown are means ± SE (n = 5). Different lower case letters in each column indicate statistically significant differences between treatments for the same species, and different capital letters in each row, significant differences between species for each treatment, according to Tukey test (α = 0.05).

| NaCl (mM) | Species | | | |
| --- | --- | --- | --- | --- |
|  | *L. santapolense* | *L. virgatum* | *L. girardianum* | *L. narbonense* |
| 0 | 1.3 ± 0.1aB | 1.0 ±0.1aA | 1.4 ± 0.1aB | 1.3 ± 0.1aB |
| 200 | 10.7 ± 0.3bB | 9.5 ± 0.3bA | 9.4 ± 0.2bA | 9.3 ± 0.3bA |
| 400 | 17.4 ± 1.0cA | 16.8 ± 0.6cA | 16.5 ±0.9cA | 17.2 ± 0.4cA |
| 800 | 27.5 ± 0.7dA | 30.5 ± 0.5dB | 28.1 ± 0.5dA | 32.5 ± 1.2dC |
